# High Fructose Diet inducing diabetes rapidly impacts olfactory epithelium and behavior in mice

**DOI:** 10.1038/srep34011

**Published:** 2016-09-23

**Authors:** Sébastien Rivière, Vanessa Soubeyre, David Jarriault, Adrien Molinas, Elise Léger-Charnay, Lucie Desmoulins, Denise Grebert, Nicolas Meunier, Xavier Grosmaitre

**Affiliations:** 1 CNRS, UMR6265 Centre des Sciences du Goût et de l’Alimentation, F-21000 Dijon, France; 2 INRA, UMR1324 Centre des Sciences du Goût et de l’Alimentation, F-21000 Dijon, France; 3 Université de Bourgogne-Franche Comté, UMR Centre des Sciences du Goût et de l’Alimentation, F-21000 Dijon, France; 4 INRA, UR1197 Neurobiologie de l’Olfaction et Modélisation en Imagerie, Domaine de Vilvert, F-78350 Jouy-en-Josas, IFR 144 Neuro-Sud Paris, France; 5 Université de Versailles Saint Quentin en Yvelines, F-78000 Versailles, France

## Abstract

Type 2 Diabetes (T2D), a major public health issue reaching worldwide epidemic, has been correlated with lower olfactory abilities in humans. As olfaction represents a major component of feeding behavior, its alteration may have drastic consequences on feeding behaviors that may in turn aggravates T2D. In order to decipher the impact of T2D on the olfactory epithelium, we fed mice with a high fructose diet (HFruD) inducing early diabetic state in 4 to 8 weeks. After only 4 weeks of this diet, mice exhibited a dramatic decrease in olfactory behavioral capacities. Consistently, this decline in olfactory behavior was correlated to decreased electrophysiological responses of olfactory neurons recorded as a population and individually. Our results demonstrate that, in rodents, olfaction is modified by HFruD-induced diabetes. Functional, anatomical and behavioral changes occurred in the olfactory system at a very early stage of the disease.

Olfactory sensory neurons (OSNs) represent the first element of the olfactory system. They convert the chemical information of odorants into electrical information integrated by the brain. Several factors can modulate peripheral olfactory neurons among which the environment and metabolic state.

Several receptors of the metabolic peptides and hormones involved in the nutritional status are largely expressed in the olfactory epithelium^1^,^2^,^3^,^4^,^5^,^6^. These receptors and hormones will take part in the modulation of odor detection by the nutritional status. Among these actors, insulin and leptin vary with both nutritional state and diet. They modulate the olfactory system and olfactory-driven behaviors^4^,^5^,^7^,^8^,^9^. So far, there is little data on the effect of diet at the peripheral level of the olfactory system. An hyperlipidemic diet generating metabolic syndrome induces anatomical, functional changes in the olfactory mucosa as well as behavioral alterations^10^. Besides this study, the influence of metabolic disorders on olfaction has been rarely explored. Among them, diabetes, especially type 2 diabetes (T2D), represents a major public health issue since it reaches worldwide epidemic level^11^,^12^. In humans, T2D is associated with poorer olfactory performance at the level of identification, discrimination or detection threshold^13^,^14^,^15^. In most experiments, the state of T2D and the potential role of secondary pathologies (such as diabetic neuropathy) are rarely mentioned. The effect of diabetes in itself and especially early diabetes on olfaction is unknown.

Overconsumption of fructose is now considered a major contributor to T2D development^16^ and HFruD is largely used to induce T2D in rodents^17^,^18^. Thus, in this study, we expose mice to a fructose enriched diet (HFruD for High Fructose Diet) inducing diabetes after 4 to 8 weeks. We then observe the consequences of this diet on the peripheral olfactory system as well as on olfactory behaviors. We find that overconsumption of fructose rapidly induces structural and functional changes in the peripheral olfactory system. Amplitudes of electro-olfactogram responses to odorants are reduced while their kinetics are modulated. Cell dynamics are impaired with a reduced apoptosis level and an increased density of mature neurons. Using patch-clamp recordings on individual olfactory sensory neurons, we show that HFruD decreases OSNs’ responses to odorant stimulation associated with reduced excitability and transduction pathway capacity. Finally HFruD strongly disrupts olfactory behaviors: diabetic mice are unable to discriminate between 2 odorants and are slower to find food in a test based on olfactory cues. Our results show that early diabetic state induced by HFruD dramatically disrupts olfaction.

## Results

### HFruD elicits functional impairment of olfactory epithelium

First we confirmed that the High Fructose Diet (HFruD) used here can induce type 2 diabetes. Mice were fed with HFruD for 4 or 8 weeks. As expected, the HFruD progressively induced diabetes phenotype with fasting hyperglycemia, hyperinsulinemia and glucose intolerance (Supplementary Fig. 1). These effects appeared as early as after 4 weeks of diet and persisted after 8 weeks of diet.

**Figure 1:**
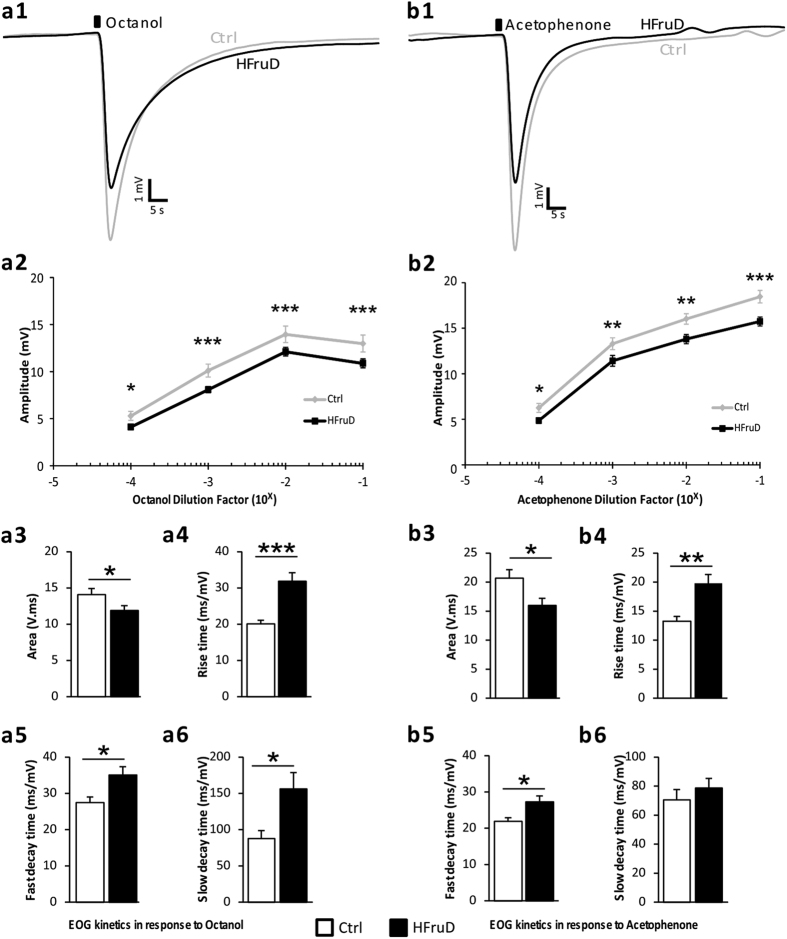
EOG responses are decreased after 8 weeks of HFruD. Global responses to octanol (**a**) or acetophenone (**b**) were recorded from olfactory mucosa of control (grey lines and white bars, n = 22 recordings from 11 mice) and HFruD (dark lines and bars, n = 32 recordings from 16 mice) animals. (1) Representative EOG traces after odorant stimulation (dark square) at 1:1000 dilution in mineral oil. (2) Amplitudes of EOG responses. Lines represent mean amplitudes values (±SEM) for different concentrations of odorants. *p < 0.05, **p < 0.01 and ***p < 0.001 after two-ways ANOVA followed by Fisher’s LSD post-hoc tests. (3–6) EOG kinetics at 1:1000 dilution in mineral oil. Bar graphs represent mean values (±SEM). **p < 0.01, ***p < 0.001 after Student’s t test. (3) Area under curve. (4) Rise time (time needed between 10 and 90% of the maximum response). (5) Fast decay time (between 100 and 80% of the maximum response). (6) Slow decay time (between 40 and 20% of the maximum response).

To assess the physiological consequences of HFruD on the peripheral olfactory system, electrical activity of olfactory epithelium in response to odorant stimulations was measured using air-phased electro-olfactogram (EOG) as described earlier^19^,^20^. Responses to octanol and acetophenone were recorded after 4 (Supplementary Fig. 2) and 8 (Fig. 1) weeks of diet. Amplitudes of EOG responses were measured for concentrations of odorants ranging from 1:10000 to 1:10 vol/vol dilution. EOG kinetics were measured on the responses to stimulations with the 1:1000 concentration of odorants.

After 4 weeks of diet, EOG amplitudes in response to octanol decreased in HFruD animals (p = 0.06, Supplementary Fig. 2a2) only for the highest concentration. No difference in EOG kinetics was observed for octanol responses (Supplementary Fig. 2a3–6). In response to acetophenone, EOG amplitudes were decreased in HFruD animals (p < 0.01, Supplementary Fig. 2b2) for all concentrations except 1:10,000. The responses to acetophenone were slower since (i) the rise time increased by a factor 1.27 (p < 0.01, Supplementary Fig. 2b4) and (ii) both fast and slow decay times increased by a factor of 1.37 (p < 0.001, Supplementary Fig. 2b5) and of 1.57 (p < 0.01, Supplementary Fig. 2b6) respectively.

After 8 weeks of HFruD, the reduction of EOG amplitudes was amplified and generalized to the 2 odorants tested: EOG were strongly reduced for both octanol (p < 0.001, Fig. 1a2) and acetophenone (p < 0.001, Fig. 1b2) at all concentrations. All parameters of the responses’ kinetics were also modified by HFruD: the responses were smaller and slower. The area under the curves was reduced for stimulations by both octanol (by 15%, p < 0.05, Fig. 1a3) and acetophenone (23%, p < 0.05, Fig. 1b3). Furthermore, rise time, slow and fast decay times were higher for HFruD animals after stimulation by octanol (by factors of, respectively: 1.58, p < 0.001; 1.28, p < 0.05; 1.78, p < 0.05; Fig. 1a4). For acetophenone stimulation, rise time and fast decay time increased in HFruD mice compared to control mice (by factors of, respectively: 1.49, p < 0.01; 1.25, p < 0.05; Fig. 1b4).

To summarize, after 8 weeks of diet, EOG amplitudes were reduced and EOG kinetics were slower in HFruD animals compared to control animals. After 4 weeks of HFruD, EOG responses show intermediate changes: they were slower in HFruD animals compared to control only for responses to acetophenone. These results could be explained by changes in the cell dynamics in the olfactory mucosa and/or functional impairment at the level of individual neurons.

### HFruD alters cell dynamics in the olfactory mucosa (OM)

**Figure 2:**
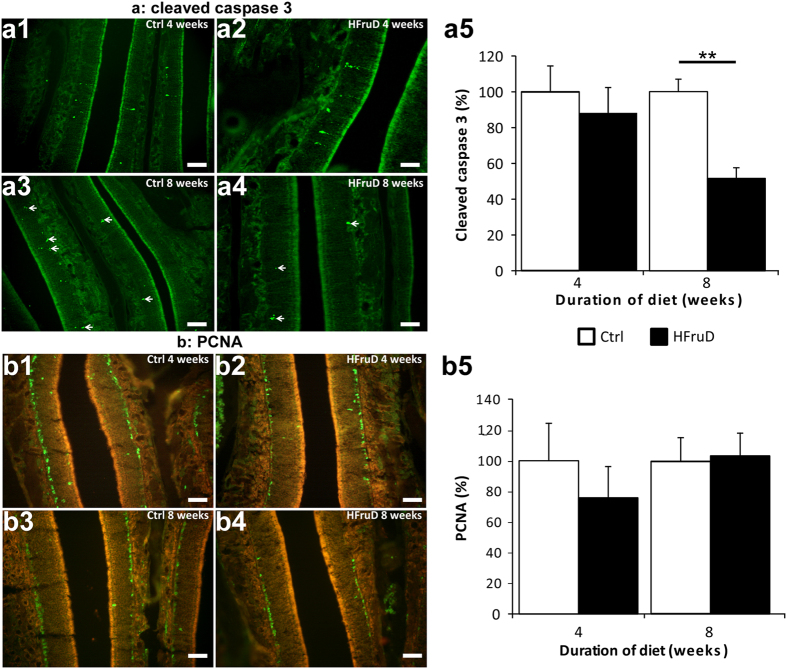
Apoptosis is reduced in olfactory mucosa after 8 weeks of HFruD. Bar graphs represent relative quantification (mean ± SEM) of fluorescent labelling for control and HFruD animals. (**a**) Apoptosis labelling using an antibody against cleaved caspase 3. (a1–4) Representative cleaved caspase 3 labelling (green) images. Scale bars: 50 μm. (a5) Quantification of relative fluorescence of cleaved caspase 3 labelling after 4/8 weeks of diet (Control n = 4/7 animals, HFruD n = 6/8 animals). **p < 0.01 after Mann-Whitney’s test. (**b**) Cell proliferation labelling using an antibody against PCNA. (b1–4) Representative PCNA labelling (green) images. Scale bars: 50 μm. (b5) Quantification of relative fluorescence of PCNA labelling after 4/8 weeks of diet (control n = 5/7 animals, HFruD n = 6/7 animals).

Cell dynamics in the olfactory epithelium is characterized by a constant regeneration of OSNs from proliferating basal cells and a subtle control of the apoptosis/proliferation balance^21^. Here apoptosis level in the olfactory mucosa was assessed by immunohistochemistry using an antibody against cleaved caspase 3 (Fig. 2a). After 4 weeks of diet, no difference in the cleaved caspase 3 labeled area was observed between control and HFruD animals. However, after 8 weeks of diet, HFruD animals displayed a 48.1% decrease in cleaved caspase 3 signal in the olfactory mucosa compared to control animals (p < 0.001, Fig. 2a3). A cell counting quantification procedure confirmed these results: 8 weeks of HFruD induces a 49.0% reduction of the number of caspase 3 labeled cells compared to control animals (p = 0.00146, see Supplementary Fig. 3a). In order to further confirm these results, we measured apoptosis using DeadEnd^TM^ Fluorometric TUNEL system after 8 weeks of HFruD. Using this technique, a 53% decrease in apoptosis was also measured in HFruD animals compared to control animals (p < 0.05, Supplementary Fig. 3b). Proliferation of basal cells was assessed by immunohistochemistry using an antibody against PCNA (for Proliferating Cell Nuclear Antigen), a classical proliferating cell marker^22^. No difference between control and HFruD animals was found either after 4 or 8 weeks of diet (Fig. 2b).

**Figure 3:**
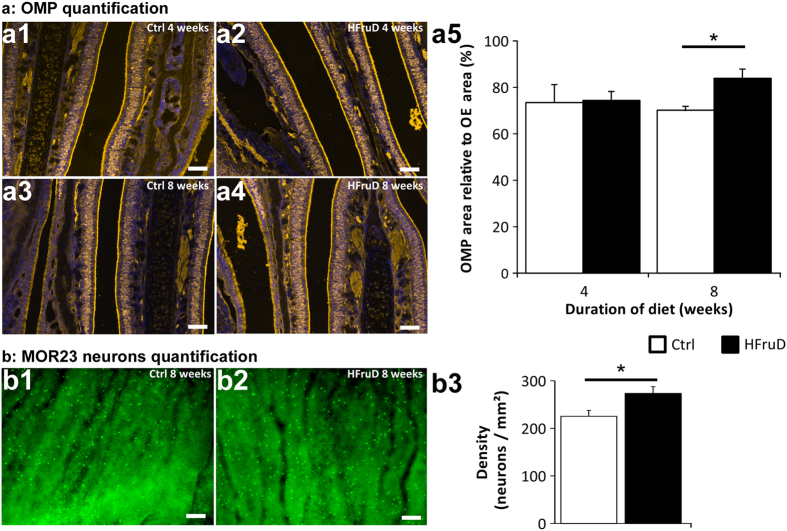
Olfactory sensory neurons’ number is increased after 8 weeks of HFruD. (**a**) The number of OSNs in the olfactory mucosa was determined using an antibody against OMP labelling. (a1–4) Representative OMP labelling images. Yellow: OMP, blue: Hoechst. Scale bars: 50 μm. (a5) Quantification of relative OMP labelling area after 4/8 weeks of diet (control n = 5/6 animals, HFruD n = 5/6 animals). *p < 0.05 after Mann-Whitney’s test. (**b**) Whole-mount preparation of olfactory epithelium showing MOR23 neurons’ density increase after 8 weeks of HFruD. (b1–2) Representative images of MOR23 neurons expression in the olfactory epithelium. Scale bar: 50 μm. (b3) Quantification of MOR23 neurons’ density in the olfactory epithelium. Bar graphs represent mean values (±SEM) for control (n = 12) and HFruD (n = 9) animals. *p < 0.05 after Mann-Whitney’s test.

What are the consequences of this disruption of cell turnover in the OM? The number of mature OSNs was measured by immunohistochemistry using an antibody against OMP (Fig. 3a). To quantify the number of mature neurons, we measured relative areas of OMP-labelling (see methods). No difference in relative OMP area was found after 4 weeks of diet between control and HFruD animals. However, after 8 weeks of diet, HFruD animals displayed a 20% increase in OMP-labelling area (p < 0.05, Fig. 3a3). Meanwhile the thickness of the olfactory mucosa did not vary between control and HFruD mice whether at 4 weeks or at 8 weeks of diet (Supplementary Fig. 3c), We also measured the density of MOR23 expressing neurons using a flat mount preparation of septal olfactory epithelium after 8 weeks of HFruD. For the MOR23 population of OSNs, a 17.59% increase of OSNs’ density was measured in HFruD animals compared to control animals (p < 0.05, Fig. 3b). These results are consistent with the increase in OMP staining in HFruD animals compared to control animals. Reconstructions of cilia on MOR23 neurons revealed no difference in the number and the length of their cilia (Supplementary Fig. 3d). These data show that HFruD elicits an increase in the number of neurons, especially mature neurons in the OM.

### HFruD reduces the sensitivity and modulates the kinetics of the odorants’ responses in individual OSNs

**Figure 4:**
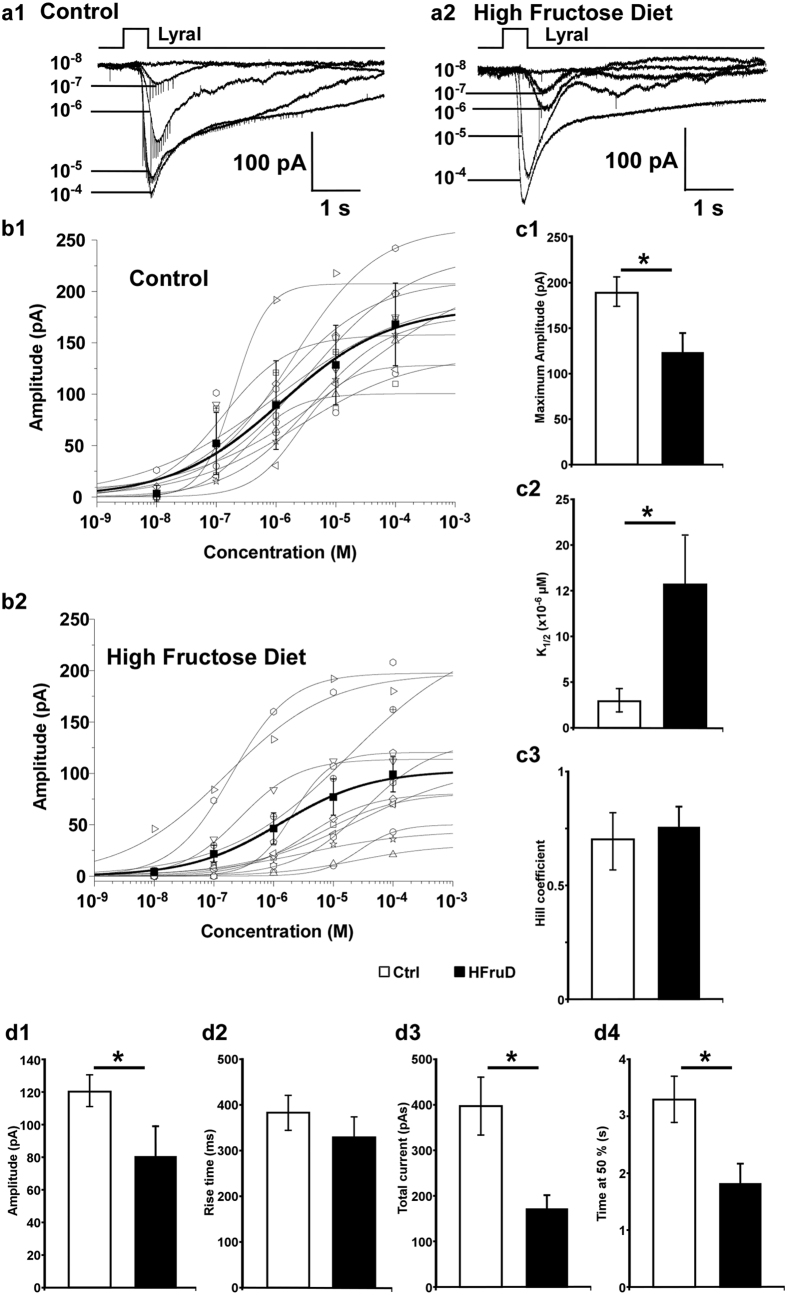
Eight weeks of HFruD reduces the sensitivity and changes the kinetics of odorant induced responses in MOR23 neurons. (**a**) Dose–response relationships of MOR23 cells in response to Lyral: inward currents induced by Lyral at different concentrations (indicated by the numbers in mol/l) under voltage clamp with one example of a control cell (a1) and a HFruD cell (a2). (**b**) The dose–response curves of the peak currents from control (n = 11 cells from 6 mice, b1) and HFruD (n = 12 cells from 6 mice, b2). The black symbols and thick black line correspond to the average (±SEM) of the control or HFruD neurons. (**c**) Quantification of the dose-response characteristics: bar graphs representing the average maximum amplitude (c1), the average K1/2 the concentration at which half of the maximum response was reached (c2) and the average Hill coefficient (c3). (**d**) Quantification of the kinetics characteristics of control and HFruD MOR23 neurons in response to 10^−5^ M Lyral: analysis of the maximum amplitude (d1, control: n = 16, HFruD: n = 10), the rise-time (d2, control: n = 17, HfruD: n = 10), the total current elicited (d3, control: n = 16, HFruD: n = 10) and the time at 50% (d4, control: n = 17, HFruD: n = 10). All recordings performed in perforated patch and at a membrane potential of −67mV. Data represented as mean ± SEM; *p < 0.05 after Student’s t test.

In order to monitor the effect of HFruD on the odorant elicited responses in individual OSNs, we performed patch-clamp recordings on MOR23 expressing neurons after 8 weeks of diet. These recordings were performed on the dendritic knob of GFP labeled neurons in an intact preparation as described previously^19^,^23^. In voltage clamp mode, all MOR23 neurons respond to increasing Lyral concentration stimuli with inward currents showing increasing maximum amplitude. These responses were observed in both control (Fig. 4a1) and HFruD (Fig. 4a2) mice. The peak transduction currents versus the concentration are plotted and fitted with the Hill equation: I = Imax/(1+(K_1/2_/C)^n^), where I represents the peak current, Imax the maximum response at saturating concentrations, K_1/2_ the concentration at which half of the maximum response was reached, C the concentration of odorant and n the Hill coefficient (Fig. 4b). HFruD induced changes in the dose-response characteristics of MOR23 neurons. First, the responses were smaller since the average maximum amplitude elicited by saturating concentrations was reduced by 35% in HFruD mice compared to control mice (p < 0.05, Fig. 4c1). Second, the HFruD neurons were less sensitive to Lyral since the dose-responses were shifted to the right: the K_1/2_ increased from 3.0 μM ± 1 in control cells to 15.6 μM ± 5 (p < 0.05, Fig. 4c2) in HFruD cells. Besides, the population of HFruD neurons exhibits a much larger variability than control neurons: a one tail F-test shows that the variance of the HFruD group is significantly larger than the variance of the control group (F = 0.047, p = 0.000017). However, there was no effect of HFruD on the dynamic range of the responses since the Hill coefficient in HFruD or control mice did not differ significantly (Fig. 4c3). These results indicate that HFruD induces a reduction of sensitivity and of maximum responses of MOR23 expressing OSNs.

We also noticed that the shape of the responses differed in HFruD cells compared to control cells. In fact, inward currents in HFruD neurons were characterized by a smaller amplitude, and a slower return to the baseline compared to control neurons. We therefore analyzed the kinetics of the responses for a specific concentration of Lyral, 10^−5^ M, close to the K_1/2_. All MOR23 neurons tested responded at this concentration. The kinetics parameters changed between HFruD and control mice. First, the maximum amplitude dropped by 34% between control and HFruD neurons (p < 0.05, Fig. 4d1). Second, the responses’ lengths were shorter since the time at 50% decreased by 45% in HFruD mice compared to control mice (p < 0.05, Fig. 4d4). Third, the total current elicited was also 57% smaller in HFruD mice compared to the total current in control mice (p < 0.05, Fig. 4d3), further indicating that the responses were shorter. Meanwhile the responses’ onset was not faster since the rise time did not change significantly (Fig. 4d2).

To further monitor the total capacity of their transduction pathway, we stimulated MOR23 OSNs with a mixture of 200 μM IBMX and 20 μM of forskolin. The maximum amplitude of the inward current elicited by IBMX + forskolin mixture was 46% smaller in HFruD mice (108.0 pA ± 26.8; n = 9) compared to control mice (199.8 pA ± 32.2; n = 11, p < 0.05). This result indicates that HFruD induces a reduction in the transduction machinery’s capacity in MOR23 neurons.

Does HFruD also affect other membrane properties of MOR23 neurons such as firing properties and excitability? We first recorded their spontaneous firing activity in the current clamp configuration during 30 to 40 s epochs. There was no difference between control and HFruD neurons neither for the average firing frequency, nor for the instantaneous firing frequency (Supplementary Fig. 4a). Then the excitability of the neurons was measured by the injection of a 7 pA depolarizing current over 2 s epochs. The 7 pA current induced action potentials in both groups but HFruD neurons exhibited a lower excitability (Supplementary Fig. 4b): (i) the depolarizing current induced 53% less action potentials in HFruD neurons compared to control neurons (p < 0.05); (ii) the latency (time between the onset of the stimulating current and the first spike) increased by a factor 1.42 in HFruD neurons compared to control neurons (p < 0.01). Meanwhile, the interspike interval did not change (p = 0.33). The reduced excitability of HFruD neurons was not due to changes in the resting membrane potential since we did not observe differences for this parameter between control (−64.5 mV ± 2, n = 8) and HFruD neurons (−65.9 mV ± 2, Z = 0.48, p = 0.91, KS-test).

These results show that, after 8 weeks of diet, HFruD induced in MOR23 neurons a reduction of the response to the ligand of the MOR23 receptor. This reduction may be due to a lower transduction pathway capacity associated with a lower excitability.

### HFruD induces impairment of olfactory abilities

**Figure 5:**
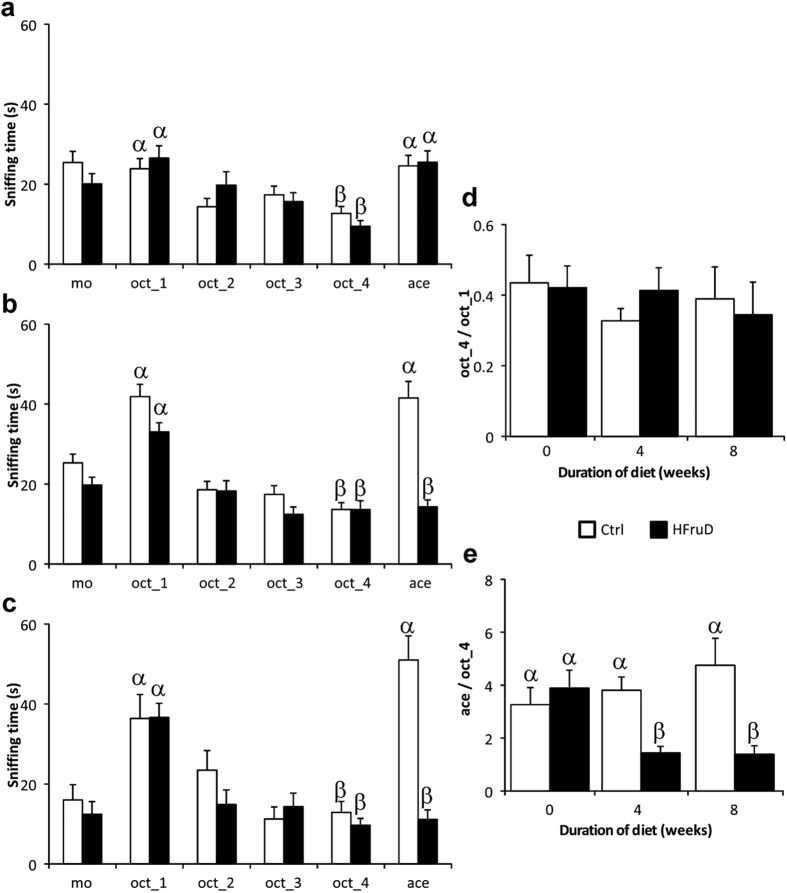
Olfactory discrimination is impaired after HFruD. Habituation/Dishabituation tests were performed before (**a**) and after 4 (**b**) and 8 weeks (**c**) of diet in control (n = 28) and HFruD (n = 28) animals. (**a–c**) Habituation/Dishabituation tests. Bar graphs represent the mean sniffing time (±SEM) of animals. The sniffing time is measured during six successive trials of 3 minutes (mineral oil, 4 times octanol, acetophenone). (**d,e**) Bar graphs represent mean value (±SEM) for habituation and dishabituation ratios. (**d**) Habituation ratio (last octanol trial on first octanol trial). (**e**) Dishabituation ratio (acetophenone trial on last octanol trial). Values with different superscripts differ significantly (α ≠ β) (two-ways ANOVA followed by Fisher’s LSD post-hoc tests).

Olfactory abilities were measured in animals using several behavioral experiments. First, the ability of animals to differentiate between two unfamiliar odorants was assessed using the habituation/dishabituation test. Animals were tested longitudinally: before any change in their diet (Fig. 5a) and then after 4 (Fig. 5b) and 8 weeks of HFruD (Fig. 5c). The ability to differentiate between two odorants is characterized by a normal habituation and dishabituation^24^. In our experimental set-up, we exposed four times the animals to octanol then to acetophenone. We observed a similar habituation and a similar dishabituation in both groups before the change of diet (Fig. 5a, p = 0.86 with two way ANOVA).

After 4 weeks of diet, HFruD animals displayed altered sniffing time compared to control animals (p < 0.01 with two way ANOVA). Both control and HFruD animals displayed normal habituation. However only control animals displayed increased sniffing time for dishabituation (Fig. 5a): the sniffing time between the last octanol trial and the acetophenone trial increased in control animals (p < 0.001) while remained constant in HFruD animals (p = 0.93). After 8 weeks of diet, both control and HFruD animals still displayed habituation. Again only control animals displayed increased sniffing time for dishabituation (p < 0.001) while HFruD animals did not show dishabituation (p = 0.6). Statistical significance values are summarized on the Supplementary Table S1A.

To monitor longitudinally our two groups of animals, two ratios were calculated: a habituation ratio (last trial with octanol on first trial with octanol, Fig. 5d) and a dishabituation ratio (trial with acetophenone on last trial with octanol, Fig. 5e). Habituation was not modified by HFruD (p = 0.27 with two way ANOVA) but dishabituation was clearly reduced for HFruD animals compared to control animals (p < 0.05 with two way ANOVA): only HFruD animals displayed a decreased dishabituation ratio between 0 and 4 or 8 weeks of diet. Furthermore, dishabituation ratio of HFruD animals was 62% smaller than that of control animals after 4 weeks of diet (p < 0.01) and 70% smaller after 8 weeks of diet (p < 0.001, Fig. 5e).

To make sure that these results were due to an inability to differentiate octanol and acetophenone and not just to a decrease in detection of acetophenone in HFruD animals, the same test was performed on a different group of animals reversing the order of presentation of the two odorants: here acetophenone was used as the habituation odorant and octanol as the dishabituation odorant. The same pattern of results was found by doing the test this way, i.e. an impairment of HFruD animals to perform the habituation/dishabituation test (Supplementary Fig. 5 and Supplementary Table S1B). These results indicate that HFruD clearly reduces the olfactory discrimination in mice. This reduction takes place already after 4 weeks of diet and is maintained after 8 weeks.

**Figure 6:**
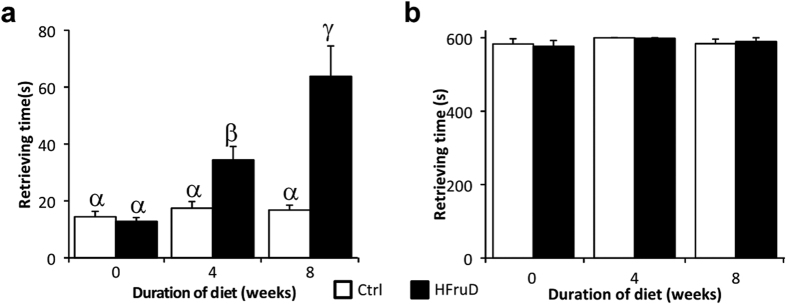
HFruD mice display reduced olfactory abilities for a food odor. Buried food behavioral tests were performed on control and HFruD animals before and after 4 and 8 weeks of diet. Bar graphs represent the mean time (±SEM) for retrieving food or control items for control (n = 17) and HFruD (n = 17) animals. (**a**) Retrieving time for a food item. (**b**) Retrieving time for a control item. Values with different superscripts differ significantly (α ≠ β ≠ γ) (two-ways ANOVA followed by Fisher’s LSD post-hoc tests).

General olfactory abilities for a food odor were assessed using the buried food test, in which we measured the time needed to retrieve a food or control item buried under the bedding (Fig. 6). Animals were tested before and after 4 to 8 weeks of diet to do longitudinal follow up of each animal. Decreased olfactory abilities were found in HFruD animals compared to control animals (p < 0.001 with two way ANOVA, Fig. 6). More precisely the time needed to retrieve the food item did not change for control animals throughout time, i.e. between 0 and 4 or 8 weeks of diet. By contrast HFruD animals displayed an 2.7 fold increase in the time needed to retrieve a food item between 0 and 4 weeks (p < 0.01) and a 5 fold increase between 0 and 8 weeks of diet (p < 0.001). The time to retrieve the food item is therefore 2 to 3 times higher in HFruD mice compared to control mice after 4 to 8 weeks. In the meantime, the time to retrieve a control item was not modified by the HFruD (p = 0.82 with two way ANOVA). In summary, HFruD rapidly induces a strong reduction of the ability to detect and find a food item.

Several non-olfactory factors can also explain those behavioral results. To test whether HfruD animals were simply less motivated to forage for food, the same experiment was performed but this time with a visible food item simply placed on the bedding. After 8 weeks of HFruD the time needed to retrieve the food item was not different between control and HFruD animals (p = 0.64, Supplementary Fig. 6b).

The two behavioral tests are also dependent on locomotor activity and anxiety of animals. To test whether these parameters are affected by HFruD, open field experiments were performed before and after 4 to 8 weeks of diet (Supplementary Fig. 6a). No effect of HFruD was found on locomotor activity measured by total walking distance and average speed (respectively p = 0.52 and p = 0.47 with two way ANOVA). No effect of HFruD was found on anxiety assessed using thigmotaxis, a natural behavior of mice which avoid the center of an open arena (p = 0.54 with two way ANOVA).

In summary, our results show that olfactory behaviors are rapidly and profoundly disrupted in mice under HFruD.

## Discussion

Here we report that a High Fructose Diet rapidly induces early diabetic state in mice and modifies the peripheral olfactory system. First, HFruD induces changes in the cell dynamics in the olfactory epithelium. Second, HFruD disrupts the peripheral responses to odorants: we observe reduced amplitude and kinetics of the EOG associated with reduced sensitivity and transduction pathway capabilities in individual OSNs. HFruD, inducing early diabetes, strongly disrupts olfaction: animals are not able to discriminate between 2 odorants and need more time to retrieve a food item in a test based on olfactory cues.

We observe an increase of the number of mature OSN (Fig. 3). Meanwhile, the amplitude of the EOG responses to odorants decreases (Fig. 1). This result seems counterintuitive as while neurons mature from P0 to P30, their sensitivity increases^25^. However, we also observe that individual OSNs exhibit a reduced sensitivity and transduction pathway capacity (Fig. 4) and this result is consistent with the decreased response recorded in EOG. One hypothesis might be that the mature neurons observed in HFruD mice are in fact aging neurons with reduced transduction properties. The population of recorded OSNs shows a large variability in their sensitivity supporting this hypothesis: the OSN population is more diverse with newborn and mature neurons as well as more aging neurons.

In HFruD animals, the apoptosis level in the OM is lower compared to control animals (Fig. 2). This lower level of apoptosis in HFruD mice compared to control of the same cohort is consistent with a higher number of MOR23 OSNs (Fig. 3). This lower level of apoptosis is also consistent with the increased OMP stained OM area in HFruD mice. Indeed, even if its quantification can be biased due to its important expression, this marker is expressed by mature neurons^26^,^27^ and it should be more present if apoptosis level is decreased. While the number of mature neurons increases, the thickness of the olfactory epithelium did not change (Supplementary Fig. 3). These results suggest that the number of other cell types, whether immature neurons or supporting cells, might decrease under HFruD. Further investigations will provide more information about these cell types and the mechanism involved in changing the balance between mature neurons and other cell types during cell proliferation. One hypothesis to explain the change in the apoptosis level induced by HfruD is based on the action of insulin in the OM. Indeed, after 4 to 8 weeks of HFruD, animals present a phenotype of early diabetic state with hyperinsulinemia and hyperglycemia (Supplementary Fig. 1). At this stage, pancreatic beta cells generate a large amount of insulin inducing fasting hyperinsulinemia (compensation period of diabetes^28^). Since insulin is known to be an anti-apoptotic factor for olfactory epithelium cells *in vivo*^29^, the hyperinsulinemia may in turn induce a reduction of apoptosis in the OM. As diabetes advances, the level of insulin drops^30^. It could be of interest to follow the consequences of advanced diabetes on the OM’s dynamics. In our conditions, diabetes alone is sufficient to rapidly disrupt cell dynamics, but long term consequences of diet induced diabetes on cell dynamics may probably be even more dramatic. These long term effects may however be due to secondary complications that appear later in the disease^31^. Another model of long term application (6 months) of Western diet induces obesity^10^. This long term metabolic disorder paradigm induces a reduction of the number of mature neurons with increased apoptosis and proliferation. However no hyperglycemia or hyperinsulinemia was observed in these conditions suggesting that this increase in apoptosis would be due to obesity. Our data show that early diabetic phenotype is sufficient to induce anatomical changes in the olfactory system.

Could hyperglycemia induce cell dynamic changes? Hyperglycemia induces apoptotic changes in dorsal root ganglion (DRG) neurons and Schwann cells both *in vivo* and *in vitro*^32^,^33^,^34^. It seems therefore doubtful that, in our model, hyperglycemia would reduce apoptosis. Modulation of cell dynamics could potentially involve Neuropeptide Y (NPY) since NPY may be involved in the regulation of apoptosis and microvillar cells in the epithelium^35^. However NPY levels in HFruD mice are unknown. Finally, at this stage of diabetes, we can exclude diabetic neuropathy since neuropathy primarily starts on large cells and appears late in the development of diabetes in humans^31^. In rodents, 16 weeks of HFruD is not long enough to develop diabetic neuropathy^36^. Hyperinsulinemia remains the main hypothesis for HFruD effects on the cell dynamics in the OM.

HFruD rapidly induces a reduction of the sensitivity of the olfactory epithelium observed through EOGs (Fig. 1 and Supplementary Fig. 2). Both amplitude and kinetics are modulated by HFruD, indicating that the transduction pathways of OSNs are modulated in our conditions. Interestingly, long term High Fat diet induces reduction in amplitude of EOGs without changes in the EOG kinetics^10^ associated with a reduction of mature neurons. The effects of short term diabetes and long term metabolic disorder might therefore involve different pathways. We also report that diabetes induced by 8 weeks of HFruD modulates functional properties of individual OSNs: the odorant responses’ amplitude and also the sensitivity of OSNs are reduced (Fig. 4). This lower sensitivity to odorant stimulus is associated with a reduction of both the excitability (Supplementary Fig. 4) and the total transduction pathway capacities of OSNs. Such functional changes could be triggered by modifications of the cilia’s structure^37^. In our conditions however, no modification of length or number of OSNs’ cilia was observed (Supplementary Fig. 3b) suggesting other mechanisms such as a modulation of transduction pathway proteins’ transcription levels. Both chronic hyperglycemia and hyperinsulinemia may explain the modulation of OSNs’ activity. However, to our knowledge, there is no report of OSNs glucose sensing properties that might modulate their activity. On the other hand, rats EOGs and OSNs’ activity can be modulated by insulin^2^,^8^. However, these results show that *acute* application of insulin reduces EOG amplitude and increases the spontaneous activity and excitability of OSNs. Here we possibly observe the effects of *chronic* hyperinsulinemia leading to insulin resistance. Chronic hyperinsulinemia may have consequences on the OSNs properties through the insulin transduction pathway. Both MAPK and PI3K-Akt pathways could be activated by insulin and lead to gene regulation of transduction pathway proteins (for review, see refs 38 and 39). Finally, hyperinsulinemia and hyperglycemia are known to modulate mitochondria’s properties^40^,^41^. Since mitochondria are fundamental for shaping the OSNs’ responses^42^, the impact of chronic hyperglycemia and hyperinsulinemia on OSNs’ mitochondria will need to be investigated further: i.e. the expression of mitochondrial proteins, mitochondrial trafficking or ATP production.

HFruD profoundly disrupts olfactory behaviors, whether during a discrimination task (Fig. 5) or during olfactory based food searching task (Fig. 6). These behavioral effects could be explained by anxiety disorders. It was recently shown that HFruD does not induce depressive disorders in mice and has no effect on anxiety-related behaviors^43^. Our data also confirmed that anxiety is not changed in HFruD animals (Supplementary Fig. 6). Consequently, anxiety and/or depression related disorders cannot explain our observations. Consequently, our data show an effect of HFruD on olfactory capacities. Our observation of functional impairment at the periphery fits well with the behavioral data. However, behavioral alterations will definitely also be due to changes at more central levels. For instance, the olfactory bulb will be modulated by HFruD since it is extremely sensitive to insulinemia and glycemia^44^,^45^. The entire OB network and more specifically mitral cells should be modulated^46^. Here too, the role of chronic hyperinsulinemia could in part explain our behavioral results. Indeed, insulin icv injection in the lateral ventricle decreases olfactory-driven behaviors for both food and non-food odors^9^.

Are HFruD mice hungrier than control mice? Hunger is often associated with type 2 diabetes in Humans. However, this is not always the case when using a HFruD in mice: for instance, food intake is not modified by the HFruD^47^,^48^. The visible cheese experiment presented here (Supplementary Fig. 6) takes place with lights on: mice can therefore easily locate the food source using visual and olfactory cues. Consequently, the latency to grab the food is more related to motivation of the animal to eat. There is no difference between HFruD and control mice for retrieving the visible food item. This data fits well with the absence of difference in food intake^47^,^48^ to show that HFruD mice are not hungrier. Results presented here are therefore the consequence of olfactory dysfunction.

Our data reinforce observations in diabetic patients showing a reduction of olfactory capacities^13^,^14^,^15^. In human subjects however, it remains difficult to separate the primary role of diabetes and the role of secondary complications such as neuropathy. Some studies even proposed that olfactory dysfunctions in diabetic patients are due to, or at least aggravated by, secondary pathologies^13^,^14^,^15^. In our experimental paradigm, animals exhibit an early diabetic phenotype. This phenotype is therefore sufficient to disrupt olfactory behavior and physiology. Therefore, it would be possible that olfactory dysfunctions during the onset of T2D and after the appearance of secondary pathologies rely on different mechanisms. Since olfaction plays a major role in regulating food intake, its early modulation could aggravate diabetic phenotypes through deregulation of feeding behaviors that may in turn intensify the T2D symptoms.

## Methods

### Histology

#### Flat mount preparation

Septal olfactory epithelia were dissected in a Ringer solution. One part of them was mounted in a perfused chamber and visualized using an Olympus BX51WI microscope (40X objective) coupled to a PCO Imaging SensiCam camera. An extra 2x magnification was achieved by a magnifying lens in the light path. Images were analyzed with FIJI and OSNs’ cilia were reconstructed using the FIJI plugin Simple Neurite Tracer. Differences in number and length of cilia were analyzed using a Mann-Whitney’s test with Statistica software. The other part of septal epithelia was then processed and neurons observed in a flatmount epithelium as published earlier^19^. The neuronal density was calculated as the ratio of the number of GFP-containing neurons against surface identified as the area including all visible GFP containing neurons. Differences in the neuronal density were analyzed using a Mann-Whitney’s test with Statistica software.

#### Olfactory epithelium’s sections

Olfactory epithelium sections were obtained as published earlier^20^. For staining, sections were incubated either with a mouse anti-PCNA antibody (1/100, GeneTex), a rabbit anti-cleaved caspase 3 antibody (1/400, Ozyme) or a goat anti-OMP antibody (1/500, Wako). Sections were then incubated with, respectively, a goat anti-mouse Alexa Fluor 488 antibody (1/1000, molecular probes), a goat anti-rabbit Alexa Fluor 488 antibody (1/1000, molecular probes) or a donkey anti-goat Alexa Fluor 555 antibody (1/1000, molecular probes). Alexa Fluor 488 (green) was used since, in our experimental conditions, at the end of the fixation and decalcifications steps, olfactory epithelia did not exhibit any remaining intrinsic GFP fluorescence (Supplementary Fig. 7a). Cell nuclei were labelled using Hoechst staining (1/5000, molecular probes). TUNEL staining was performed in accordance with the manufacturer’s instructions (DeadEnd^TM^ Fluorometric TUNEL system, Promega) and a final Hoechst staining was performed. For cleaved caspase 3, PCNA and TUNEL staining, results were expressed in relative expression of fluorescent staining compared to total olfactory epithelium area as performed earlier^29^,^49^,^50^. Apoptosis and proliferation were quantified by the area of signal rather than the number of cells^49^,^50^ to overrule the difficulty of counting very fragmented cells for caspase 3 staining and overlapping stained cells for PCNA. The apical area of the epithelium, which contains only cilia and no cell body, was excluded from the region of interest for quantification because of its particularly strong autofluorescence (Supplementary Fig. 7b). PCNA, TUNEL and C3C results were expressed as a relative value of the control group. To further validate the relevance of the fluorescence quantification, C3C labeling was also quantified by cell counting (see results on Supplementary Fig. 3a; for details, see Supplemental methods). For OMP staining, total area of OMP labelling was quantified and expressed in relative values of total olfactory epithelium area. This OMP area is correlated with the number of OMP-positive cells (r^2^ = 0.91, p < 0.05, data not shown). All statistical analysis were Mann-Whitney’s tests performed with Statistica software. All images were taken and all analyses were performed blindly of the experimental groups.

#### Electro-olfactogram

EOG recordings were performed from the olfactory mucosa in an opened nasal cavity on mouse hemi-heads as described earlier^20^. Mice hemi-heads were stimulated with the same odorants used for behavioral experiments: octanol and acetophenone. EOG voltage signals were analyzed using Clampfit 9.2 (Axon Instruments) to measure responses’ peak amplitude, area under curve, rise time, fast and slow decay times. Rise time and two decay times were normalized to the corresponding response peak amplitude prior to statistical analysis. For responses to various concentrations of odorants, two way ANOVAs followed by Fisher’s LSD post hoc tests were performed. For kinetics, Student’s t tests were performed. All statistical analyses were performed with Statistica software.

### Patch-clamp recordings

Patch-clamp recordings were performed as described earlier^19^,^23^,^51^. Olfactory epithelia were harvested from MOR23-IRES-tauEGFP mice and perfused with Ringer’s solution, which contained (in mM): NaCl 124, KCl 3, MgSO_4_ 1.3, CaCl_2_ 2, NaHCO_3_ 26, NaH_2_PO_4_ 1.25, glucose 15; pH 7.6 and 305 mOsm. Perforated patch-clamp was performed by including 260 μM nystatin in the recording pipette, which was filled with the following solution (in mM): KCl 70, KOH 53, methanesulfonic acid 30, EGTA 5, HEPES 10, sucrose 70; pH 7.2 (KOH) and 310 mOsm. OSNs were stimulated with pressure-delivered odorants^52^. Lyral was prepared in 0.5 M solution in dimethyl sulfoxide (DMSO) and kept at −20 °C. Forskolin was prepared in 10 mM stock solution in DMSO; IBMX was prepared in 100 mM stock solution in DMSO. Final solutions of odorants IBMX/forskolin were prepared before each experiment by adding Ringer. All chemicals were obtained from Sigma-Aldrich. Lyral was provided as a generous gift from International Fragrances and Flavors (Dijon, France). Analysis was performed using Fitmaster, SpAcAn add-on for Igor Pro^53^ and Origin. Statistical analysis (Student’s t tests, F-test) was performed using Origin software (OriginLabs).

### Behavioral experiments

#### Habituation/Dishabituation test

The olfactory habituation/dishabituation test^54^, is used to test whether the animal can differentiate different odors. Here we modified a protocol from Yang and Crawley^24^ using a special apparatus (adapted from^55^) composed of a test area of 40 × 40 × 10 cm (length × width × height), with a hole in its center. Odorant was placed under litter in the hole. Animals were submitted to 6 consecutive trials of 3 minutes: 100 μl of mineral oil; 4 trials habituation (100 μl odorant 1: octanol) and dishabituation trial (100 μl odorant 2: acetophenone). Animals were filmed during the test (conducted in red lights). Movies were analyzed offline using a custom Matlab routine, which measures the number of visits and the length of each visit in the odorized hole: the mouse position was tracked to score these parameters. This process is completely automated; however a manual scoring was performed to exclude visits where the back of the animal (and not its head) is above the odorized hole. Mean visit durations were used for analysis.

#### Open field test

Mice were placed in the same apparatus without hole. Videos were processed using a custom Matlab routine that tracked mouse centroid positions during 5 min. Open field test scoring was completely automated. Total walking distance, average speed and thigmotaxis (expressed as the percentage of total time spent close to the walls) were measured.

#### Buried food test

We modified a protocol from Yang and Crowley^24^ in which we chose Leerdammer^®^ cheese as the food stimulus instead of a chocolate. Food access was removed 5 hours before the experiment. Animals were placed in a conventional mouse cage (33 × 19 × 13 cm) filled with 6 cm of bedding. Food or control item was buried at one randomly chosen spot (out of 8) in the cage. Test ended once either the animals hold the item or did not achieve to find it within 10 minutes. Ability of animals to retrieve a visible food item on the bedding was tested in a conventional rat style cage (42 × 26 × 19 cm). The criterion to stop the experiment was when the animal started to eat the cheese.

During data analysis of all behavioral assays, experimenters were blind to the experimental groups.

All statistical analyses (using Statistica software) consisted in two way ANOVA followed by Fischer’s LSD post hoc tests except for the visible cheese experiment which was analyzed using a Student’s t test.

## Additional Information

**How to cite this article**: Rivière, S. *et al*. High Fructose Diet inducing diabetes rapidly impacts olfactory epithelium and behavior in mice. *Sci. Rep.* **6**, 34011; doi: 10.1038/srep34011 (2016).

## Supplementary Material

Supplementary Information
