# Supplementary material for: High Fructose Diet inducing diabetes rapidly impacts olfactory epithelium and behavior in mice
Source: Sci Rep. 2016 Sep 23;6:34011. doi: 10.1038/srep34011 (PMC5034277; doi:10.1038/srep34011)
Supplement: Supplementary Information [file srep34011-s1.pdf]

<u>Supplementary Information</u>

Title: High Fructose Diet inducing diabetes rapidly impacts olfactory epithelium and behavior in mice.

Authors : Sébastien Rivière, Vanessa Soubeyre, David Jarriault, Adrien Molinas, Elise Léger-Charnay, Lucie Desmoulins, Denise Grebert, Nicolas Meunier, Xavier Grosmaitre

Supplementary Figures:

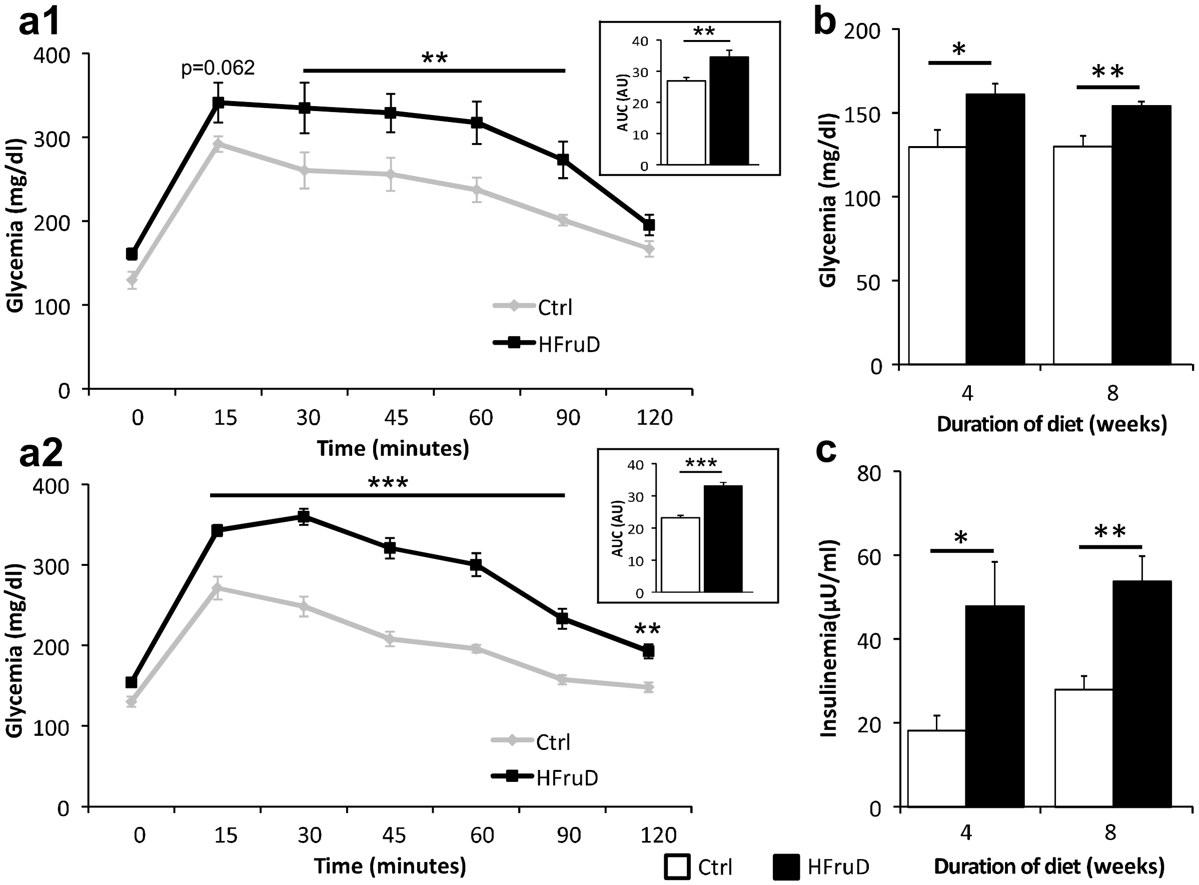

Supplementary Figure 1: Mice fed with a HFruD during 4 to 8 weeks display an early type 2 diabetes phenotype. (a) Glucose intolerance was assessed using ipGTT for control (grey lines and white bars) and HFruD (dark lines and bars) animals. Values are expressed as mean (± SEM). **: p < 0.01 and ***: p < 0.001 (two-ways ANOVA followed by Fisher's LSD post-hoc tests).Square bar graph: Area under curve. **: p < 0.01 after Mann-Whitney's test and ***: p < 0.001 after Student's t test. (a1) ipGTT after 4 weeks of diet for control (n = 9) and HFruD (n = 9) animals. (a2) ipGTT after 8 weeks of diet for control (n = 11) and HFruD (n = 13) animals. (b) Fasted glycemia. Graph bars represent mean glycemia values (± SEM) after 4 / 8 weeks of diet (Control n = 9 / 11, HFruD n = 9 / 13). (c) Fasted insulinemia. Graph bars representing the mean insulinemia values (± SEM) after 4 / 8 weeks of diet (control n = 6 / 11, HFruD n = 6 / 12). (b-c) *: p < 0.05 after Mann-Whitney's test and **: p < 0.01 after Student's t test.

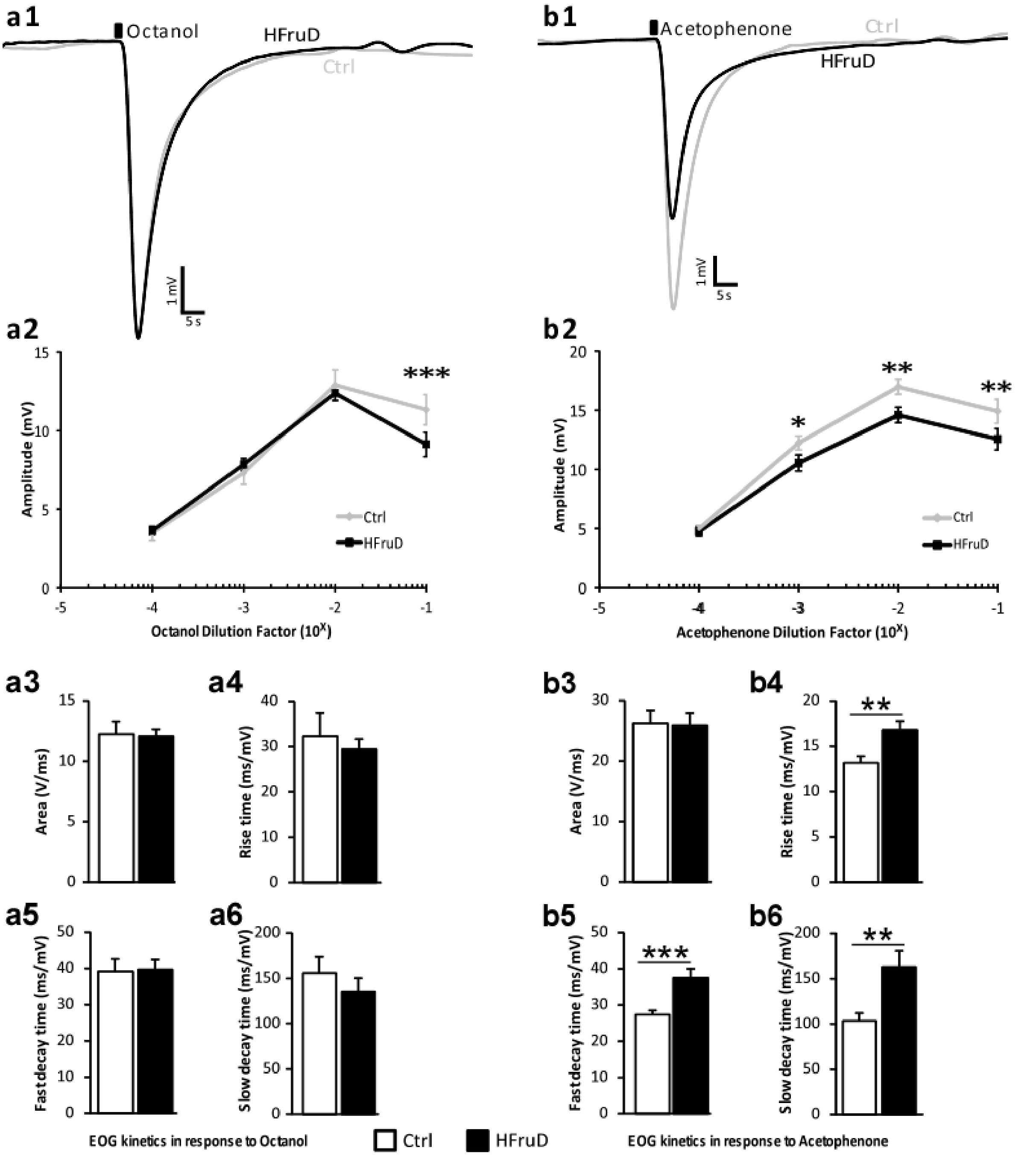

Supplementary Figure 2: EOG responses are partially reduced after 4 weeks of HFruD. Global responses to octanol (a) or acetophenone (b) were recorded from olfactory mucosa of control (grey lines and white bars, n = 16 recordings from 8 mice) and HFruD (dark lines and bars, n = 16 recordings from 8 mice) animals. (1) Representative EOG traces after odorant stimulation (dark square) at 1:1000 dilution in mineral oil. (2) Amplitudes of EOG responses. Lines represent mean amplitudes values (±SEM) for different concentrations of odorants. *: $p < 0.05$, **: $p < 0.01$ and ***: $p < 0.001$ after two-ways ANOVA followed by Fisher's LSD post-hoc tests. (3-6) EOG kinetics at 1:1000 dilution

in mineral oil. Bar graphs represent mean values (±SEM). ***: $p < 0.001$ after Student's t test. (3) Area under curve. (4) Rise time (time needed between 10 and 90% of the maximum response). (5) Fast decay time (between 100 and 80% of the maximum response). (6) Slow decay time (between 40 and 20% of the maximum response).

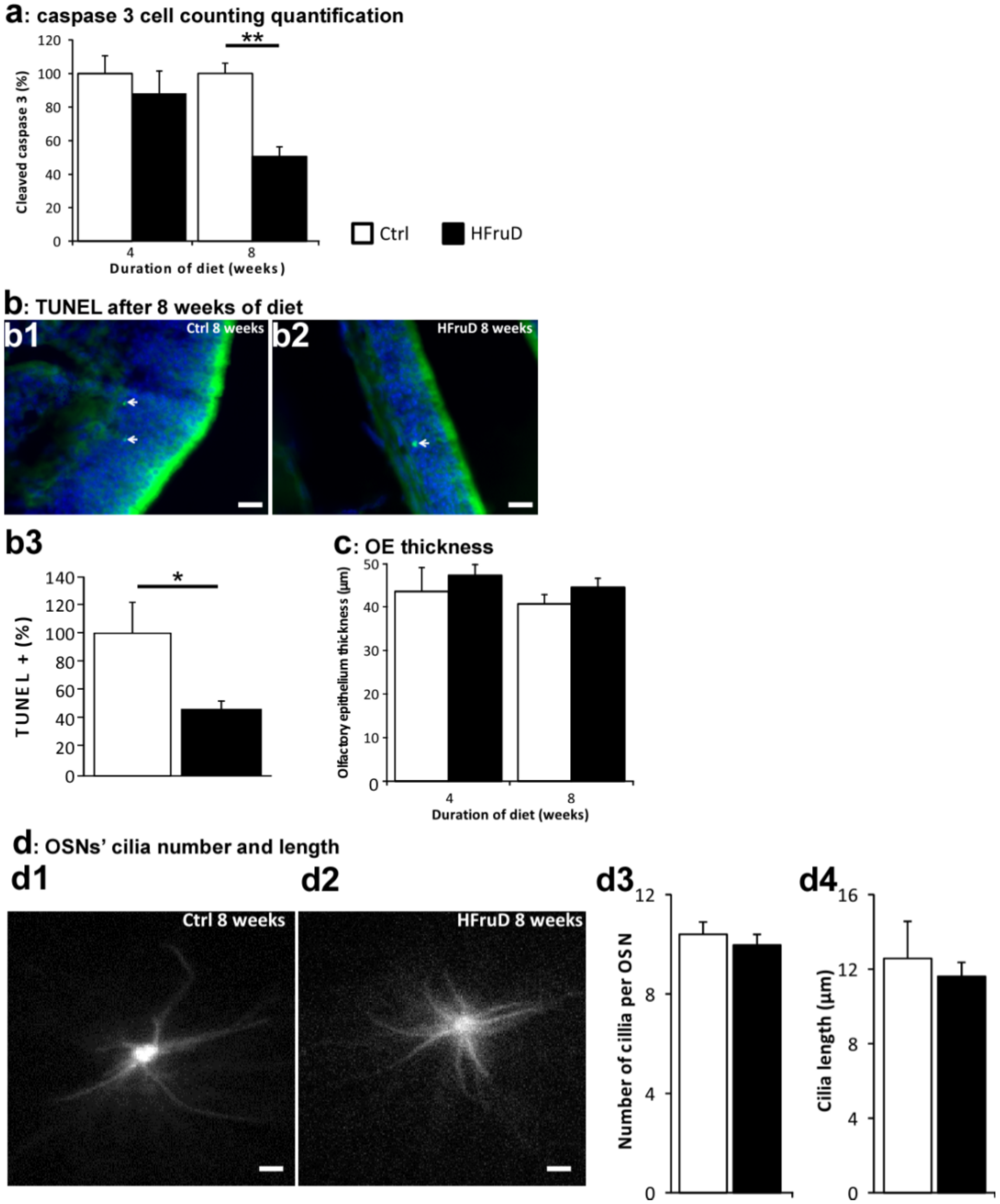

Supplementary Figure 3: Anatomical effects of HFruD on the MOE and OSNs: caspase 3 cell counting quantification, TUNEL, OE thickness and olfactory cilia properties. (a) Quantification of relative cell counting of cleaved caspase 3 labelling after 4 / 8 weeks of diet (Control n = 4 / 7 animals, HFruD n = 6 / 8 animals). **: $p < 0.01$ after Mann-Whitney's test. (b) Apoptosis detection after TUNEL staining. (b1-2) Representative TUNEL staining images. Green: TUNEL staining, blue:

Hoechst staining. Scale bars: 25 µm. (b3) Quantification of relative fluorescence of TUNEL staining for control (n = 6) and HFruD (n = 6) animals after 8 weeks of diet. *: $p < 0.05$ after Mann-Whitney's test. (c) Quantification of the thickness of the olfactory epithelium after 4 / 8 weeks of diet (control n = 5 / 6 animals, HFruD n = 6 / 7 animals). (d) Effect of 8 weeks of HFruD on MOR23 neurons's cilia characteristics (d1-2) Representative images of dendritic knobs with cilia of MOR23 neurons. Scale bars: 2 µm. (d3-4) Quantification of the number (d3) and length (d4) of olfactory cilia per OSN for Ctrl (n = 7) and HFruD (n = 7) neurons. Data are expressed as mean (± SEM).

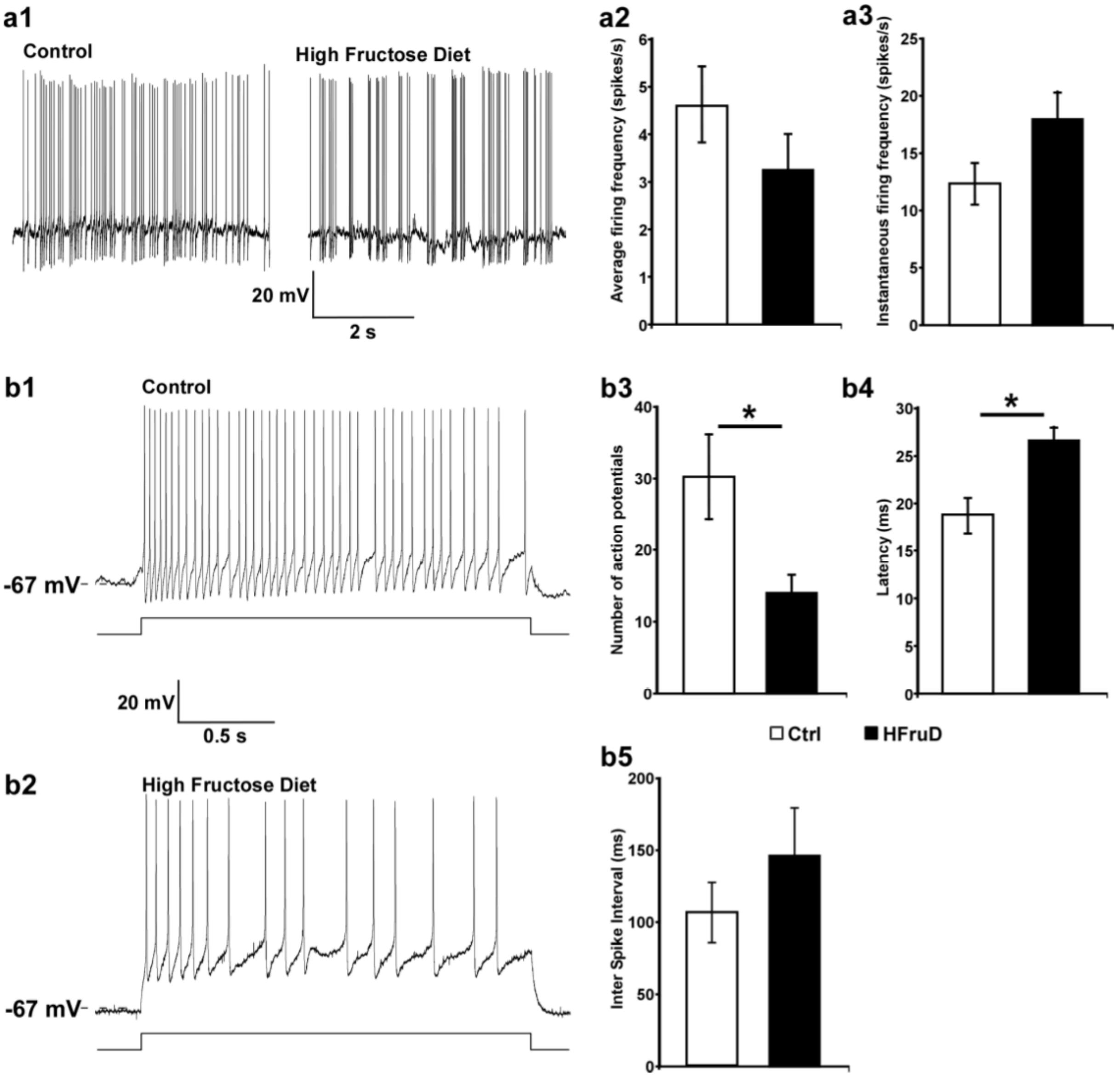

Supplementary Figure 4: Eight weeks of HFruD reduces the excitability of MOR23 neurons but does not change their spontaneous activity. (a1) Examples of spontaneous activity recorded in a control (left) and a HFruD (right) neuron. (a2-3) Quantification of the spontaneous activity recorded in control (n = 16) and HFruD (n = 11) MOR23 neurons. (b1-2) Representative examples of action potentials elicited by a 7 pA excitatory current in a control (b1) and a HfruD (b2) MOR23 neuron. (b3-5) Quantification of the excitability in control (n = 13) and HFruD (n = 18) MOR23 neurons: bar graphs representing the number of action potentials elicited by 7pA current (b3), the latency between the onset of the stimulus and the first spike (b4) and the average interval interspike of the spike train elicited (b5). All recordings performed in perforated patch and at a membrane potential of -67mV.

Data represented as mean ± SEM for neurons from control (white bars) and HFruD (dark bars) animals. *: $p < 0.05$ after Student's t test.

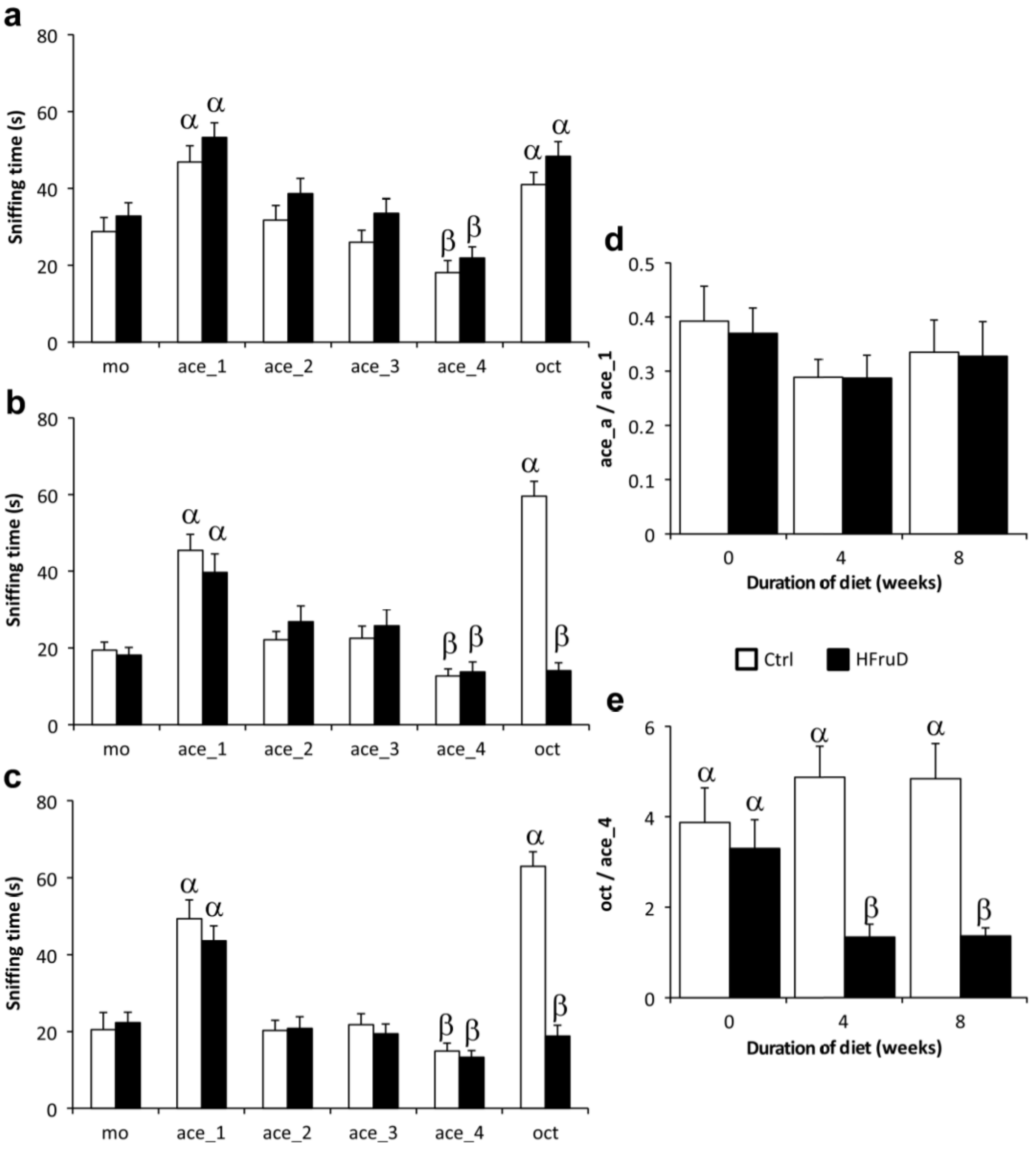

Supplementary Figure 5: Olfactory discrimination is decreased in HFruD animals, no matter the order of presentation of odorants. Habituation/Dishabituation tests were performed before (a) and after 4 (b) and 8 weeks (c) of diet in control (white bars, n = 20) and HFruD (dark bars, n = 23) animals. (a-c) Habituation/Dishabituation tests. Bar graphs represent the mean sniffing time (± SEM) of animals. The sniffing time is measured during six successive trials of 3 minutes (mineral oil, 4 times acetophenone, octanol). (d-e) Bar graphs represent mean value (± SEM) for habituation and dishabituation ratios. (d) Habituation ratio (last acetophenone trial on first acetophenone trial). (e)

Dishabituation ratio (octanol trial on last acetophenone trial). Values with different superscripts differ significantly ($\alpha \neq \beta$) (two-ways ANOVA followed by Fisher's LSD post-hoc tests).

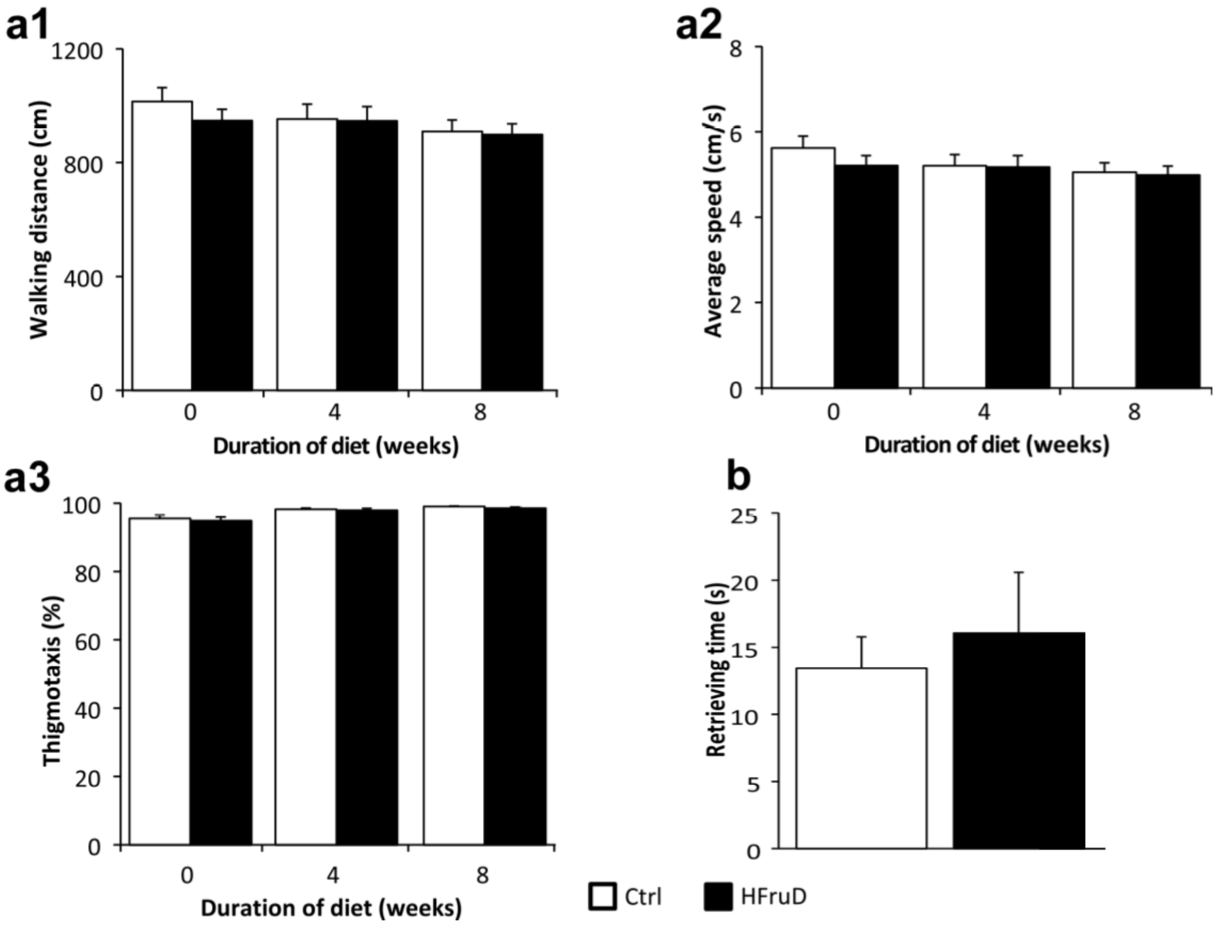

Supplementary Figure 6: Control experiments for behavioral tests. (a) Open field experiments were performed before and after 4 and 8 weeks of diet. Bar graphs represent mean values for control (white bars, n = 11) and HFruD (dark bars, n = 19) animals. Two successive trials of 5 minutes were performed for each animal. (a1) Mean walking distance. (a2) Average speed. a1 and a2 represent locomotor activity of animals. (a3) Thigmotaxis, an anxiety feature, is measured for all animals. (b) Control for the buried food experiment. Mean retrieving time for visible cheese is measured after 8 weeks of diet for control (n = 10) and HFruD (n = 13) animals.

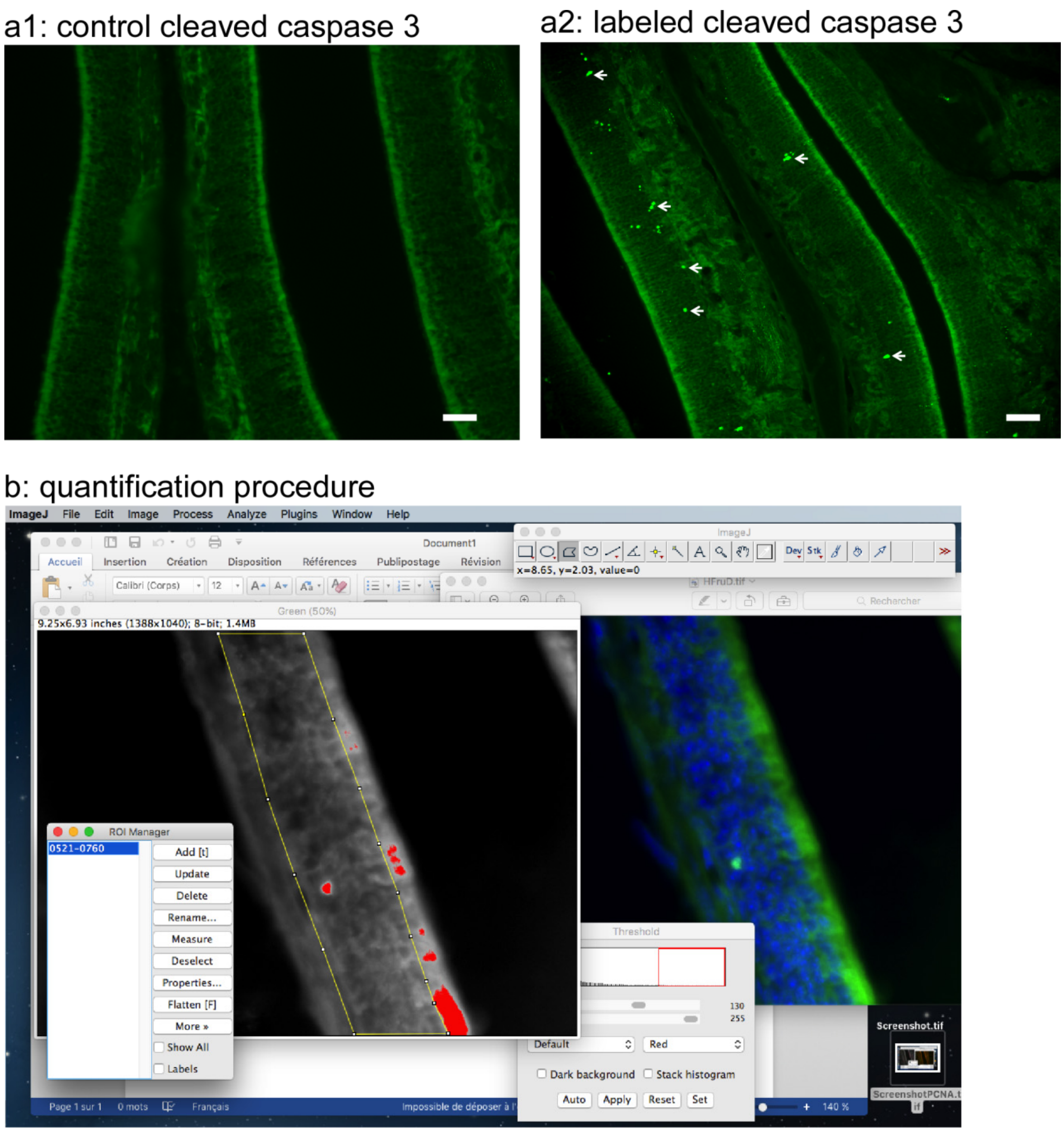

Supplementary Figure 7: Negative control showing the absence of intrinsic GFP and example of area quantification procedure. (a) Absence of intrinsic GFP in the zone 1 of the MOE after immunohistochemistry treatments: (a1) Representative image of control cleaved caspase 3 image obtained without antibody: no intrinsic GFP fluorescence can be observed; (a2) Representative image of cleaved caspase 3 labelled with antibody: green cleaved caspase 3 labelling can be seen (examples pointed by arrows). Scale bars: 50 µm. Images where taken in the zone 1 of the MOE. (b) example of quantification procedure for TUNEL experiment showing the tracing of the Region of Interest (left),

the pixel threshold set and the raw image (right); the region of interest doesn't include the autofluorescent cilia layer where there are no cell bodies.

Suppl.Table 1A: statistical values of comparison tests, habituation/dishabituation experiment with octanol as habituating odorant and acetophenone as dishabituating odorant; related to Figure 5

Comparison test	Before change of diet (Figure 5A, p values)	After 4 weeks of diet (Figure 5 B, p values)	After 8 weeks of diet (Figure 5C, p value)
oct1 ctrl *vs* oct1 HFruD	0.74	0.17	0.77
oct4 ctrl *vs* oct4 HFruD	0.32	0.83	0.78
ace ctrl *vs* ace HFrud	0.53	<0.001	<0.001
oct1 ctrl *vs* oct4 ctrl	<0.001	<0.001	<0.001
oct1 HFruD *vs* oct4 HFruD	<0.001	<0.001	<0.001
oct4 ctrl *vs* ace ctrl	<0.001	<0.001	<0.001
oct4 HFruD *vs* ace HFruD	<0.001	0.93	0.6

Suppl.Table 1B: statistical values of comparison tests, habituation/dishabituation experiment with acetophenone as the habituating odorant and octanol as dishabituating odorant; related to Supplementary Figure 5

Comparison test	Before change of diet (Suppl. Fig 5A, p values)	After 4 weeks of diet (Suppl. Fig 5B, p values)	After 8 weeks of diet (Suppl. Fig. 5C, p values)
ace1 ctrl *vs* ace1 HFruD	0.13	0.35	0.33
ace4 ctrl *vs* ace4 HFruD	0.33	0.36	0.82
oct ctrl *vs* oct HFrud	0.27	<0.001	<0.001
ace1 ctrl *vs* ace4 ctrl	<0.001	<0.001	<0.001
ace1 HFruD *vs* ace4 HFruD	<0.001	<0.001	<0.001
ace4 ctrl *vs* oct ctrl	<0.001	<0.001	<0.001
ace4 HFruD *vs* oct HFruD	<0.001	0.3	0.31

Supplementary Table 1: Statistical p values for habituation/dishabituation comparison tests. Each p value is the result of the comparison test following the two-way ANOVA and the Fisher's LSD post-hoc tests.

Detailed Methods

Animals and diets. Gene-targeted *MOR23-IRES-tauGFP* male mice were used in all experiments. Those mice express the fluorescent protein GFP in a subset of olfactory sensory neurons under the promoter of the *mor23* odorant receptor gene [1]. Mice were kept 2 or 3 per cage (conventional style cages) with food and water *ad libitum* and under constant conditions of temperature (22 ± 0.5°C) and humidity (50 ± 5%) in a 12 h light / 12 h dark inverted cycle (lights off at 5 a.m.) unless specified otherwise. From weaning to 5 weeks old, mice were maintained in a control diet (A04, sugar: corn starch 59.9%, Safe). Once they reach the postnatal age of 5 weeks, mice were split in two groups: one group remained fed with the control diet and the second one with an enriched fructose diet (HFruD, sugar: fructose 60%, Safe) for 4 to 8 weeks. All experiments were conducted during the dark phase of animals and food was removed an hour before the start of all experiments unless specified otherwise. All procedures were approved by the French Ministry of Higher Education and Research (reference 01286.02).

Glucose tolerance test and insulin assay. Intolerance to glucose was assessed using intraperitoneal glucose tolerance test (ipGTT) instead of oral glucose tolerance test because HFruD modifies glucose absorption at the intestinal level [2]. For this experiment, mice were placed in a non-inverted cycle and food was removed 5 hours before the beginning of the test, which took place during the light phase of animals. Glucose was then injected intraperitoneally (2g / kg body weight). Blood glucose was then determined with an Accu-Chek Performa glucometer (Roche) from blood sample collected from the tail vein, before and 15, 30, 45, 60, 90 and 120 minutes after glucose injection. Blood samples were also collected just before glucose injection to perform quantitative analysis of insulin level. Insulin serum levels were determined using an alphaLISA Human insulin immunoassay kit (Perkin Elmer). For ipGTT, a two way ANOVA was performed followed by Fisher's LSD post-hoc test. Areas under curve were analyzed using a Student's t test and glycemia and insulinemia using a Mann-Whitney's test. All statistics were performed using Statistica software.

Histology.

Flat mount preparation. Septal olfactory epithelia were dissected in a Ringer solution as described earlier [3]. One part of them was mounted in a perfused chamber and visualized using an Olympus BX51WI microscope (40X objective) coupled to a PCO Imaging SensiCam camera. An extra 2x magnification was achieved by a magnifying lens in the light path. Images were analyzed with FIJI and OSNs' cilia were reconstructed using the FIJI plugin Simple Neurite Tracer. Differences in number and length of cilia were analyzed using a Mann-Whitney's test with Statistica software. The other part of septal epithelia was immersed in a PBS solution containing 4% paraformaldehyde and 0.2% glutaraldehyde for 1h at room temperature. Epithelia were then washed with PBS and mounted between a slide and a coverslip with DAKO mounting medium. The septal epithelia were visualized using an Olympus BX51WI microscope (10X objective) coupled to an Olympus DP72 camera with a 4140 x 3096 resolution. GFP-containing neurons were observed using a GFP filter. Images covering the entire epithelium were taken and the whole tissue was reconstituted using the MosaicJ plugins of ImageJ software (National Institutes of Health) and the neuronal density was calculated as the ratio of the number of GFP-containing neurons against surface identified as the area including all visible GFP containing neurons. Differences in the neuronal density were analyzed using a Mann-Whitney's test with Statistica software.

Olfactory epithelium's sections: These experiments were performed as published earlier [4-6] For all other anatomy experiments, mice were deeply anesthetized with sodium pentobarbital. They were then perfused intracardially with 10ml of a solution of NaCl + heparin and then with 20ml of 4% paraformaldehyde. Heads were post-fixed for 2h in a 2% paraformaldehyde solution and then decalcified in a 5% EDTA solution for 2 weeks. Tissues were cryoprotected by immersion in a 30% sucrose solution for 48h. Heads were then dissected to keep only both nostril and placed in a solution of 50% OCT compound (Sakura, Tissue Tek) for 30 minutes followed by immersion in OCT for 30 minutes, and then frozen in nitrogen vapors. Sections (14 µm thick) were realized with a Leica cryomicrotome (Leica microsystems) and directly collected on slides. For PCNA staining, slides were incubated in a citrate buffer (pH = 6) at 95°C for 30 min for antigen retrieval. Non-specific staining

was blocked by incubation with 10% non-immune donkey serum (Abcam) diluted in PBS containing 2% bovine serum albumin and 0.3% Triton X-100. Sections were incubated either with a mouse anti-PCNA antibody (1/100, GeneTex), a rabbit anti-cleaved caspase 3 antibody (1/400, Ozyme) or a goat anti-OMP antibody (1/500, Wako). Sections were then incubated with, respectively, a goat anti-mouse Alexa Fluor 488 antibody (1/1000, molecular probes), a goat anti-rabbit Alexa Fluor 488 antibody (1/1000, molecular probes) or a donkey anti-goat Alexa Fluor 555 antibody (1/1000, molecular probes). Alexa Fluor 488 (green) was used since, in our experimental conditions, at the end of the fixation and decalcifications steps, olfactory epithelia did not exhibit any remaining intrinsic GFP fluorescence (see example on Supplementary Fig. 7A). Cell nuclei were labelled using Hoechst staining (1/5000, molecular probes). TUNEL staining was performed in accordance with the manufacturer's instructions (DeadEnd^{TM} Fluorometric TUNEL system, Promega) and a final Hoechst staining was performed. All slices were then mounted with mounting medium (ProLong Gold antifade reagent, molecular probes) under a coverslip. For caspase 3 and TUNEL experiments, olfactory epithelium slices were visualized using a Imager M2 fluorescent microscope (Zeiss) and 20 X or 40 X objectives. For PCNA experiments, slices were visualized using on a DMBR Leica microscope equipped with double filter (red/green) and an Olympus DP-50 CCD camera using CellF software (Olympus). For cleaved caspase 3, PCNA and TUNEL staining, results were expressed in relative expression of fluorescent staining compared to total olfactory epithelium area. Apoptosis and proliferation were quantified by the area of signal rather than the number of cells. This method was approved and validated for the olfactory epithelium in previous studies[4,6]. Indeed, cells ongoing apoptosis marked by TUNEL or cleaved caspase 3 can be anything between very fragmented (at the end of the apoptosis) and with a full cell body at the beginning of the process. In addition to this difference the staining can also be anything from weak to strong inducing bias in the cell counting. For caspase 3 and TUNEL experiments, a picture was taken for each area showing labeling. Since the caspase 3 staining was extremely sparse, in most of the cases, the value measured obtained was equal to 0. Therefore, the maximum of signal was quantified in order to be able to compare the C3C

signal among different animals. For each animal, 4 sections of the olfactory epithelium were used. Those sections were made at the same levels of the dorso-ventral axis for each animal to allow comparisons between animals and we kept the 12 highest scores among 20 images analyzed. For PCNA, 8 pictures were analyzed for each slice for all animals (24 pictures per animal) at similar antero-posterior coordinates in the nasal cavity, on each side of the septum: i) the posterior parts of the lateral and medial turbinates and ii) the lateral and medial turbinate at 50% of the nasal cavity. For each animal, the average labeling of these different zones is then calculated. All pictures were taken with the same exposure time. Labeling quantification was performed through measuring the amount of labeling above a certain threshold: each pixel was encoded at a level between and 0 and 255. Pixels above 130 (a value chosen empirically) were considered as labeling. The same threshold value was used for all pictures. Relative expression was quantified by calculating the ratio of labeled areas on the total area of the olfactory epithelium. PCNA, TUNEL and C3C results were expressed as a relative value of the control group. To further validate the relevance of the fluorescence quantification, C3C labeling was also quantified by cell counting (see results on Supplementary Fig. 3a): on the same images and sections used for fluorescent area quantification, individual caspase 3 labeled cells were counted. A cell density (number of cell divided by the surface of the olfactory epithelium) was calculated in cell/mm^2. Cell density results were then also expressed as a relative value of the control group.

For OMP staining, total area of OMP labelling was quantified at similar antero-posterior coordinates in the nasal cavity (as described above for PCNA). Area was expressed in relative values of total olfactory epithelium area. This OMP area is correlated with the number of OMP-positive cells, as previously reported[7] as well as in our conditions ($r^2 = 0.91$, $p < 0.05$, data not shown).

The olfactory epithelium thickness was measured perpendicular to the lamina propria on the septal epithelium. Four different measurements were performed on each picture, and 4 pictures were used for each animal. The average thickness was then calculated for each picture and each animal and expressed in µm.

All statistical analysis were Mann-Whitney's tests performed with Statistica software. All images were taken and all analyses were performed blindly of the experimental groups.

Electroolfactogram. EOG recordings were performed from the olfactory mucosa in an opened nasal cavity on mouse hemiheads as described earlier [8] . Mice were sacrificed by decapitation and the hemi-head was placed in a recording chamber under an upright Olympus SZ51 stereo microscope (Olympus) equipped with a low magnification objective (0.8 to 4x) and two MX-160 micromanipulators (Siskiyou, Inc.). The odor stimulation device was modified from Scott and Brierley [9]. The hemi-head was kept under a constant flow of humidified filtered air (~1000 ml/min) delivered through a 9 mm glass tube. This tube was positioned 2 cm from the epithelial surface. Odor stimulations were performed by blowing air puffs (200 ms, 200 ml/min) through an exchangeable Pasteur pipette enclosed in the glass tube containing a filter paper impregnated with 20 µL of odorant diluted in mineral oil (Sigma Aldrich). The odorants used were the same odorants used for behavioral experiments: octanol ranging from 1:10,000 to 1:10 and acetophenone ranging from 1:10,000 to 1:10. EOG voltage signals were recorded using an XtraCell two-channels amplifier (DIPSI) used in a DC current-clamp configuration (I = 0), digitized at a rate of 5 kHz using an Digidata 1322a A/D converter (Axon Instruments, Molecular Devices) interfaced to a PC equipped with Pclamp 9.2 software (Axon Instruments). A reference Ag/AgCl electrode was placed on the frontal bone overlaying the olfactory bulb. Recordings were made with glass micropipettes of 4-5MΩ filled with a saline solution. EOG were recorded from the center of turbinates IIb and III. These positions gave robust, reproducible and long-lasting EOG recordings ranging from 10 to 18 mV when stimulated with acetophenone 1:1000. Odorant-free air stimulation (with mineral oil), always produced signals around 1 mV amplitude. Analyses were performed using Clampfit 9.2 (Axon Instruments) to measure peak amplitude, area under curve, rise time (from 10% to 90%), fast (from 100% to 80%) and slow (from 40% to 10%) decay times of EOG responses. Since the EOG response kinetics highly correlate with the amplitude, the rise time and the two decay times were normalized to the corresponding response peak amplitude prior to statistical analysis. For the analysis of the amplitudes of responses

to various concentrations of odorants, two way ANOVAs followed by Fisher's LSD post hoc tests were performed. For the analysis of EOG Kinetics, Student's t tests were performed. All statistical analyses were performed with Statistica software.

Patch-clamp recordings.

Patch-clamp recordings were performed as described earlier [3,10,11]. Briefly, MOR23-IRES-tauEGFP mice were anesthetized by i.p. injection of ketamine HCl and xylazine (150 mg/kg and 10 mg/kg body weight, respectively), and then decapitated. The head was immediately put into ice cold Ringer's solution, which contained (in mM): NaCl 124, KCl 3, $MgSO_4$ 1.3, $CaCl_2$ 2, $NaHCO_3$ 26, NaH_2PO_4 1.25, glucose 15; pH 7.6 and 305 mOsm. The pH was kept at 7.4 by bubbling with 95% O_2 and 5% CO_2. The nose was dissected out *en bloc*. The olfactory epithelium attached to the nasal septum and the dorsal recess was removed and kept in oxygenated Ringer. Right before starting the recording session, the entire epithelium was peeled away from the underlying bone and transferred to a recording chamber with the mucus layer facing up. Oxygenated Ringer was continuously perfused at room temperature.

The dendritic knobs of OSNs were visualized through an upright microscope (Olympus BX51WI) equipped with an Olympus DP72 camera and a 40x water-immersion objective (numerical aperture 0.8). An extra 4x magnification was achieved by a magnifying lens in the light path. The GFP+ labeled cells were visualized under fluorescent illumination. Superimposition of the fluorescent and bright field images allowed identification of the fluorescent cells under bright field, which directed the recording pipettes. Electrophysiological recordings were controlled by an EPC-10 USB amplifier combined with Patchmaster software (HEKA Electronic, Germany). Perforated patch-clamp was performed by including 260 µM nystatin in the recording pipette, which was filled with the following solution (in mM): KCl 70, KOH 53, methanesulfonic acid 30, EGTA 5, HEPES 10, sucrose 70; pH 7.2 (KOH) and 310 mOsm. The junction potential was ~ 9 mV and was corrected in all experiments off-line. For odorant-induced transduction currents, signals were sampled at 20 kHz. Under voltage-clamp mode, the signals were initially filtered at 10 kHz and then at 2.9 kHz.

A seven-barrel pipette was used to deliver stimuli by pressure ejection through a picospritzer (Pressure System IIe, Toohey Company, Fairfield, NJ). The stimulus electrode was placed ~20 µm downstream from the recording site. Distance and pressure were adjusted in order to minimize mechanical responses [12]. All stimuli were delivered at a 138 kPa (~20 psi) pressure indicated on the picospritzer with 500 ms pulse length. Lyral was prepared in 0.5 M solution in dimethyl sulfoxide (DMSO) and kept at –20°C. Final solutions for odorants were prepared before each experiment by adding Ringer to reach the desired concentration. To assess the total capacity of the OSNs' transduction pathway, we recorded the response to a mixture of 200 µM IBMX and 20 µM of forskolin. IBMX is a potent inhibitor of phosphodiesterase [13,14], and forskolin is an activator of adenylyl cyclase [15,16]. Forskolin was prepared in 10 mM stock solution in DMSO; IBMX was prepared in 100mM stock solution in DMSO. Final solution containing 200 µM of IBMX and 20 µM of forskolin was prepared before each experiment by adding Ringer. All chemicals were obtained from Sigma-Aldrich. Lyral was provided as a generous gift from International Fragrances and Flavors (Dijon, France).

Data was analyzed using Fitmaster (HEKA) and IgorPro (Wavemetrics). Using Fitmaster, we measured the following characteristics of the responses to odorants in voltage clamp: maximum amplitude, rise time (duration in ms from 0 to 90 % of the maximum amplitude), time at 50% (duration in ms of the response's width at 50% of the maximum amplitude), total current elicited (area under the curve in pAs). Dose-response curves were drafted and fitted using Origin software (OriginLabs). In current clamp, firing frequency was analyzed using the SpAcAn add-on for Igor Pro [17]. Statistical analysis (Student's t tests, F-test) was performed using Origin software (OriginLabs).

Behavioral experiments:

Habituation/Dishabituation test: The olfactory habituation/dishabituation test [18], which relies on the animal's tendency to investigate novel smells, is used to test whether the animal can differentiate different odors. Here we modified a protocol from Yang and Crawley [19] using a special apparatus to measure the time mice spent in exploring odors. Briefly the apparatus adapted from Mandairon et al.

[20] is composed of a test area of 40 x 40 x 10 cm (length x width x height), with in its center a hole. A beaker containing a small piece of odorized paper covered by a metallic grid and 1 to 2cm of litter is placed under the hole. Mice were first train for 3 days prior testing to explore the apparatus while mineral oil alone was added on the paper. This training period is essential to reduce the exploration time of the animals placed in a novel environment [21]. On the test day, animals were submitted to 6 consecutive trials of 3 minutes. Animals were allowed to rest in a cage for 4 minutes between each trial (another mouse was performing the task in the meantime). First trial consisted in 100 µl of mineral oil; for the following four trials (habituation) 100 µl of the first odorant were used and the last trial (dishabituation) consisted in 100µl of the second odorant. Two different odorants were used as habituation or dishabituation stimulus: octanol and acetophenone (Sigma). Since the two odorants exhibit different volatility, it was important to equilibrate their vapor pressures to be able to compare the exploration times of animals. Here we used a vapor pressure of 1Pa for both odorants (1:13 for octanol and 1:43 for acetophenone in mineral oil). Animals were filmed during the test (conducted in red lights). Movies were analyzed offline using a custom Matlab routine, which measures the number of visits and the length of each visit in the odorized hole: the mouse position was tracked to score these parameters. This process is completely automated; however a manual scoring was performed to exclude visits where the back of the animal (and not its head) is above the odorized hole. Mean exploration times were used for analysis. The critical indicators of a normal ability to smell are significant habituation and dishabituation [19]. Therefore, two ratios were calculated: habituation ratio (last exploration time for the habituation odorant divided by first exploration time for the habituation odorant) and dishabituation ratio (exploration time for the dishabituation odorant divided by the exploration time for the last habituation odorant).

Open field test. Mice were placed in the same apparatus as for the habituation/dishabituation test, except that no hole was present in the test area. Mice were tested two times for 5 minutes and allowed to rest for 6 minutes between the two trials. Animals were filmed during the test (conducted in red lights). Videos were processed using a custom Matlab routine that tracked mouse centroid

positions during 5min. Open filed test scoring was completely automated. Total walking distance, average speed and thigmotaxis (expressed as the percentage of total time spent close to the walls) were measured.

Buried food test. The buried food test [22,23], which relies on the animal's natural tendency to use olfactory cues for foraging, is usually used to confirm ability to smell volatile odors. Here we modified a protocol from Yang and Crowley [19] in which we chose Leerdammer® cheese as the food stimulus instead of a chocolate cookie (HFruD animals being used to a high-sugar flavor). Animals were habituated to cheese on two consecutive days before the actual test by placing a little piece of cheese in their home cages and check that it has been eaten on the following morning. On test day, food access was removed 5 hours before the experiment. . Animals were placed in a conventional mouse cage (33 x 19 x 13 cm) filled with 6 cm of bedding. Food or control item (a pebble of equal size as the piece of cheese) was buried at one randomly chosen spot (out of 8) in the cage. Test ended once either the animals found the item or did not achieve to find it within 10 minutes. The criterion used to stop the experiment was when the animal held the item with forepaws or with teeth. Animals were tested for 3 consecutive trials (food, control then food item). Only the second trial with food was used to analyze the results. To test for motivation of animals to search for food, we tested the ability of animals to retrieve a visible food item on the bedding in a conventional rat style cage (42 x 26 x 19 cm). The criterion to stop the experiment was when the animal started to eat the cheese.

During data analysis of all behavioral assays, experimenters were blind to the experimental groups. All statistical analyses (using Statistica software) consisted in two way ANOVA followed by Fischer's LSD post hoc tests except for the visible cheese experiment which was analyzed using a Student's t test.

Supplementary references

1 Vassalli, A., Rothman, A., Feinstein, P., Zapotocky, M. & Mombaerts, P. Minigenes impart odorant receptor-specific axon guidance in the olfactory bulb. *Neuron* **35**, 681-696 (2002).

2 Tobin, V. *et al.* Insulin internalizes GLUT2 in the enterocytes of healthy but not insulin-resistant mice. *Diabetes* **57**, 555-562, doi:10.2337/db07-0928 (2008).
3 Cadiou, H. *et al.* Postnatal odorant exposure induces peripheral olfactory plasticity at the cellular level. *J Neurosci* **34**, 4857-4870, doi:10.1523/JNEUROSCI.0688-13.2014 (2014).
4 Francois, A. *et al.* Early survival factor deprivation in the olfactory epithelium enhances activity-driven survival. *Front Cell Neurosci* **7**, 271, doi:10.3389/fncel.2013.00271 (2013).
5 Lacroix, M. C. *et al.* Insulin but not leptin protects olfactory mucosa from apoptosis. *J Neuroendocrinol* **23**, 627-640, doi:10.1111/j.1365-2826.2011.02154.x (2011).
6 Laziz, I. *et al.* Endothelin as a neuroprotective factor in the olfactory epithelium. *Neuroscience* **172**, 20-29, doi:10.1016/j.neuroscience.2010.10.063 (2011).
7 Moon, C. *et al.* Leukemia inhibitory factor promotes olfactory sensory neuronal survival via phosphoinositide 3-kinase pathway activation and Bcl-2. *J Neurosci Res* **87**, 1098-1106, doi:10.1002/jnr.21919 (2009).
8 Negroni, J. *et al.* Neuropeptide Y Enhances Olfactory Mucosa Responses to Odorant in Hungry Rats. *Plos One* **7**, doi:10.1371/journal.pone.0045266 (2012).
9 Scott, J. W. & Brierley, T. A functional map in rat olfactory epithelium. *Chemical senses* **24**, 679-690 (1999).
10 Jarriault, D. & Grosmaitre, X. Perforated Patch-clamp Recording of Mouse Olfactory Sensory Neurons in Intact Neuroepithelium: Functional Analysis of Neurons Expressing an Identified Odorant Receptor. *J Vis Exp*, doi:10.3791/52652 (2015).
11 Grosmaitre, X., Vassalli, A., Mombaerts, P., Shepherd, G. M. & Ma, M. Odorant responses of olfactory sensory neurons expressing the odorant receptor MOR23: a patch clamp analysis in gene-targeted mice. *Proc Natl Acad Sci U S A* **103**, 1970-1975, doi:10.1073/pnas.0508491103 (2006).
12 Grosmaitre, X., Santarelli, L. C., Tan, J., Luo, M. & Ma, M. Dual functions of mammalian olfactory sensory neurons as odor detectors and mechanical sensors. *Nat Neurosci* **10**, 348-354, doi:10.1038/nn1856 (2007).
13 Firestein, S., Darrow, B. & Shepherd, G. M. Activation of the sensory current in salamander olfactory receptor neurons depends on a G protein-mediated cAMP second messenger system. *Neuron* **6**, 825-835 (1991).
14 Lowe, G. & Gold, G. H. Nonlinear amplification by calcium-dependent chloride channels in olfactory receptor cells. *Nature* **366**, 283-286, doi:10.1038/366283a0 (1993).
15 Sato, T., Hirono, J., Tonoike, M. & Takebayashi, M. Tuning specificities to aliphatic odorants in mouse olfactory receptor neurons and their local distribution. *J Neurophysiol* **72**, 2980-2989 (1994).
16 Sklar, P. B., Anholt, R. R. & Snyder, S. H. The odorant-sensitive adenylate cyclase of olfactory receptor cells. Differential stimulation by distinct classes of odorants. *J Biol Chem* **261**, 15538-15543 (1986).
17 Dugue, G. P., Dumoulin, A., Triller, A. & Dieudonne, S. Target-dependent use of co-released inhibitory transmitters at central synapses. *J Neurosci* **25**, 6490-6498, doi:10.1523/JNEUROSCI.1500-05.2005 (2005).
18 Gregg, B. & Thiessen, D. D. A simple method of olfactory discrimination of urines for the Mongolian gerbil, Meriones unguiculatus. *Physiol Behav* **26**, 1133-1136 (1981).
19 Yang, M. & Crawley, J. N. Simple behavioral assessment of mouse olfaction. *Curr Protoc Neurosci* **Chapter 8**, Unit 8 24, doi:10.1002/0471142301.ns0824s48 (2009).
20 Mandairon, N. *et al.* A computer-assisted odorized hole-board for testing olfactory perception in mice. *J Neurosci Methods* **180**, 296-303, doi:10.1016/j.jneumeth.2009.04.008 (2009).
21 Macknin, J. B., Higuchi, M., Lee, V. M., Trojanowski, J. Q. & Doty, R. L. Olfactory dysfunction occurs in transgenic mice overexpressing human tau protein. *Brain Res* **1000**, 174-178, doi:10.1016/j.brainres.2004.01.047 (2004).

22 Alberts, J. R. & Galef, B. G., Jr. Acute anosmia in the rat: a behavioral test of a peripherally-induced olfactory deficit. *Physiol Behav* **6**, 619-621 (1971).

23 Edwards, D. A., Thompson, M. L. & Burge, K. G. Olfactory bulb removal vs peripherally induced anosmia: differential effects on the aggressive behavior of male mice. *Behav Biol* **7**, 823-828 (1972).
